# Biogeochemical and historical drivers of microbial community composition and structure in sediments from Mercer Subglacial Lake, West Antarctica

**DOI:** 10.1038/s43705-023-00216-w

**Published:** 2023-01-30

**Authors:** Christina L. Davis, Ryan A. Venturelli, Alexander B. Michaud, Jon R. Hawkings, Amanda M. Achberger, Trista J. Vick-Majors, Brad E. Rosenheim, John E. Dore, August Steigmeyer, Mark L. Skidmore, Joel D. Barker, Liane G. Benning, Matthew R. Siegfried, John C. Priscu, Brent C. Christner, the SALSA Science Team, Carlo Barbante, Mark Bowling, Justin Burnett, Timothy Campbell, Billy Collins, Cindy Dean, Dennis Duling, Helen A. Fricker, Alan Gagnon, Christopher Gardner, Dar Gibson, Chloe Gustafson, David Harwood, Jonas Kalin, Kathy Kasic, Ok-Sun Kim, Edwin Krula, Amy Leventer, Wei Li, W. Berry Lyons, Patrick McGill, James McManis, David McPike, Anatoly Mironov, Molly Patterson, Graham Roberts, James Rot, Cathy Trainor, Martyn Tranter, John Winans, Bob Zook

**Affiliations:** 1 Department of Microbiology and Cell Science, University of Florida, Gainesville, FL USA; 2 Department of Geology and Geological Engineering, Colorado School of Mines, Golden, CO USA; 3 Center for Geomicrobiology, Aarhus University, Aarhus, DK Denmark; 4 Department of Earth and Environmental Science, University of Pennsylvania, Philadelphia, PA USA; 5 Department of Oceanography, Texas A&M University, College Station, TX USA; 6 Department of Biological Sciences, Michigan Technological University, Houghton, MI USA; 7 College of Marine Sciences, University of South Florida, St. Petersburg, FL USA; 8 Department of Land Resources and Environmental Sciences, Montana State University, Bozeman, MT USA; 9 Department of Earth Sciences, Montana State University, Bozeman, MT USA; 10 School of Earth and Environmental Sciences, University of Minnesota, Minneapolis, MN USA; 11 GFZ German Research Centre for Geosciences, Telegrafenberg, Potsdam, Germany; 12 Department of Earth Sciences, Freie Universität Berlin, Berlin, Germany; 13 Hydrologic Science and Engineering Program, Department of Geophysics, Colorado School of Mines, Golden, CO USA; 14 Polar Oceans Research Group, Sheridan, MT USA; 15 Institute for the Dynamics of Environmental Processes, University Ca’Foscari, Venice, Italy; 16 Antarctic Science Management Office, University of Nebraska, Lincoln, NE USA; 17 Applied Physics Lab, University of Washington, Seattle, WA USA; 18 School of Film and Photography, Montana State University, Bozeman, MT USA; 19 Scripps Institution of Oceanography, University of California San Diego, La Jolla, CA USA; 20 Woods Hole Oceanographic Institution, Falmouth, MA USA; 21 School of Earth Sciences, Byrd Polar and Climate Research Center, The Ohio State University, Columbus, OH USA; 22 Department of Earth and Environmental Sciences, Columbia University, New York, NY USA; 23 Department of Earth and Atmospheric Sciences, University of Nebraska, Lincoln, NE USA; 24 Film Program, Communication Studies, California State University, Sacramento, CA USA; 25 Division of Polar Life Sciences, Korea Polar Research Institute, Incheon, South Korea; 26 Department of Geology, Colgate University, Hamilton, NY USA; 27 Department of Geological Sciences and Environmental Studies, Binghamton University, Vestal, NY USA; 28 Department of Environmental Science, Aarhus University, Aarhus, Denmark; 29 Present Address: Bigelow Laboratory for Ocean Sciences, East Boothbay, ME USA

**Keywords:** Soil microbiology, Microbial ecology

## Abstract

Ice streams that flow into Ross Ice Shelf are underlain by water-saturated sediments, a dynamic hydrological system, and subglacial lakes that intermittently discharge water downstream across grounding zones of West Antarctic Ice Sheet (WAIS). A 2.06 m composite sediment profile was recently recovered from Mercer Subglacial Lake, a 15 m deep water cavity beneath a 1087 m thick portion of the Mercer Ice Stream. We examined microbial abundances, used 16S rRNA gene amplicon sequencing to assess community structures, and characterized extracellular polymeric substances (EPS) associated with distinct lithologic units in the sediments. Bacterial and archaeal communities in the surficial sediments are more abundant and diverse, with significantly different compositions from those found deeper in the sediment column. The most abundant taxa are related to chemolithoautotrophs capable of oxidizing reduced nitrogen, sulfur, and iron compounds with oxygen, nitrate, or iron. Concentrations of dissolved methane and total organic carbon together with water content in the sediments are the strongest predictors of taxon and community composition. δ¹³C values for EPS (−25 to −30‰) are consistent with the primary source of carbon for biosynthesis originating from legacy marine organic matter. Comparison of communities to those in lake sediments under an adjacent ice stream (Whillans Subglacial Lake) and near its grounding zone provide seminal evidence for a subglacial metacommunity that is biogeochemically and evolutionarily linked through ice sheet dynamics and the transport of microbes, water, and sediments beneath WAIS.

## Introduction

**Fig. 1:**
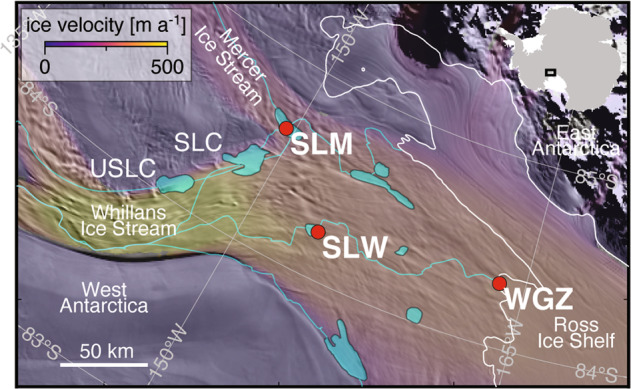
Locator map of SLM along the confluence of the Mercer and Whillans ice streams. The drilling sites for SLM, SLW, and WGZ are marked with red dots. The blue lines represent the predicted water flow paths beneath WAIS [15] and the white line indicates the ice-sheet grounding line [63]. The background imagery is MODIS MOA2009 [61] with ice velocity overlain [62].

Dynamic subglacial water systems beneath the Antarctic ice sheet are modulated by hydrologically connected subglacial lakes that episodically fill and drain, transferring water and material between basins before eventually discharging to the ocean [1–3]. Whillans Subglacial Lake (SLW; Fig. 1) is a component of that system beneath an 800 m thick portion of the West Antarctic Ice Sheet (WAIS) and was the first Antarctic subglacial lake directly sampled [4, 5]. When SLW was accessed for study in 2013, its water column was under-saturated in oxygen (∼16% of air-saturated water), had relatively high dissolved organic carbon (DOC) concentrations (~221 μmol L^−1^), and contained a diverse and metabolically active community of bacteria and archaea [6]. Rates of chemoautotrophy [6] and methanotrophy [7] were sufficient to support heterotrophic demand in the water column and agreed well with the inferred physiologies of dominant taxa identified [8], yet observed doubling times of ~200 days confirmed slow metabolisms with low growth efficiencies (8%) [6, 9]. Most biologically relevant solutes in SLW’s water column were the products of interactions among minerals, pore water, organic matter (OM), and microbes in the sediments [10]. Methane and reduced inorganic compounds may represent the most plentiful bioenergetic substrates available and can originate from processes associated with water-saturated sediments that include OM degradation [7], biotic and abiotic chemical weathering [11], and rock comminution (i.e., free radical catalyzed production of H_2_, CH_4_ and NH_4_^+^; [12]).

A 2.06 m sediment profile was recently obtained from Mercer Subglacial Lake (SLM), one of the largest subglacial lakes by area (143 km^2^) on Whillans Ice Plain (Fig. 1) and the focus of science activities for the Subglacial Antarctic Lakes Scientific Access (SALSA) project [13, 14]. Although SLM and SLW are in relatively close geographical proximity (~60 km), the lakes occupy separate hydrological basins and have differences in their recharge sources, ice thickness, and downstream-upstream lake connections [15]. A key dissimilarity between SLW and SLM is that while they receive a common source of basal melt from WAIS, modeling indicates ~21% of SLM’s volume is derived from basal melt that originates and is transported from East Antarctica [16]. SLW and SLM are positioned ~100 km from the modern ice sheet grounding zone, where freshwater that drains from upstream lakes (e.g., from Upper Conway Subglacial Lake [USLC] to Conway Subglacial Lake [SLC] to SLM; Fig. 1) is transported and discharged into the marine cavity beneath Ross Ice Shelf. During periods of WAIS grounding line retreat, regions where the modern ice sheet is grounded (i.e., the grounding zone) become inundated with seawater and transition into a floating ice shelf. Recent reports of radiocarbon-bearing sediments from Siple Coast subglacial environments have provided new results on the timing of these marine incursion events [17, 18], with data from SLM sediments indicating the last marine incursion occurred 6.3 ± 1.0 kiloyear (ka) [16]. The emerging view of WAIS dynamics during the Middle Holocene is highly relevant to studies of ecosystems and biogeochemical processes beneath the ice sheet because it means that exchange of OM and microbiota between the marine system and subglacial environment occurred more recently than previously thought [19].

We integrated a combination of biogeochemical, isotopic, microbiological, and molecular approaches to analyze the SLM composite sediment profile and test the hypothesis that microbial assemblages are differentially structured among the lithologic units and contemporary biodiversity is regulated by the mineralization of relict marine OM. We further investigated variation in and correlations of microbes and their extracellular polymeric substances (EPS) with downcore physical and geochemical gradients in the sediment column. Stable isotopic analysis of EPS-carbon was carried out to constrain the assimilatory carbon sources of microbes in the sediment. From these data, we infer compositional and biogeochemical features of the contemporary microbial community in the lake sediments. Furthermore, we analyzed microbial assemblages in sediments predating the formation of SLM (subglacial diamict), compared their similarity to those observed in sediments from the marine side of the grounding zone, and explore the possibility that they were deposited during alternating migration phases of WAIS grounding line. One advantage of the 16S rRNA gene dataset from SLM is that it facilitates direct comparison with data obtained during previous studies of SLW [6, 8] and the Whillans Grounding Zone (WGZ; [20]). A key finding from the meta-analysis is the similarity among the community structures and taxa in surficial sediments from SLM to those documented at SLW. Based on these observations and evolutionarily relatedness in microbial populations between the lakes, we examine the possibility that WAIS hosts a subglacial metacommunity that is biogeochemically and evolutionary linked through material transport and ice sheet dynamics.

## Materials and methods

### Sampling of SLM and sediment core characterization

SLM is located beneath the downstream portion of Mercer Ice Stream at its confluence with Whillans Ice Stream and was sampled at a location (84.640287° S, 149.501340° W) near the center of the lake (see the site description in the Supplementary Information). On 26 December 2018, a ~0.4 m diameter borehole made through 1087 m of ice was completed to access the lake [13] using environmentally clean hot water drilling [5, 21–23] and deployment procedures [24]. The access borehole was maintained for eight days of subglacial lake water column and sediment sampling (Supplementary Information). Information on the sediment cores and samples used in this study are provided in Table S1 and compiled data across samples are detailed in Supplementary Data File 1 based on the composite depth scale (Supplementary Information).

Whole-core computed tomography (CT) scans, magnetic susceptibility, and bulk elemental composition [i.e., derived from Geotek standard multi-sensor core logging and ITRAX X-ray fluorescence (XRF)] provided non-destructive analyses of downcore variations in SLM sediments to guide core sampling (Supplementary Information). Peaks in magnetic susceptibility, fractional porosity, and XRF data were correlated to generate a composite depth scale that stratigraphically aligned depths from all cores recovered, facilitating comparison of physical, geochemical, isotopic, and microbiological data collected in samples from the 2.06 m profile (Supplementary Information). All data are reported on a dry weight basis per gram of sediment.

### Extraction of cells and EPS from sediment

Cells were extracted and separated from sediment particles by Nycodenz density gradient centrifugation [25, 26] and enumerated via epifluorescence microscopy (Supplementary Information). EPS were extracted from sediment and water column samples in 50 mM EDTA based on previous methods [27–29] (Supplementary Information). The carbohydrate component of the EPS was quantified using UV spectrophotometry [30]; DNA that co-extracted with EPS (EPS-DNA) was quantified using the Qubit™ dsDNA HS Assay Kit (Invitrogen), and co-extracted protein was measured using the Qubit™ Protein BR Assay Kit. The methods for preparing samples of EPS for scanning transmission X-ray spectroscopy (STXM) [31–34] and excitation-emission matrices (EEMs) fluorescence spectrometry are described in the Supplementary Information.

### Geochemical and isotope analysis

**Fig. 2:**
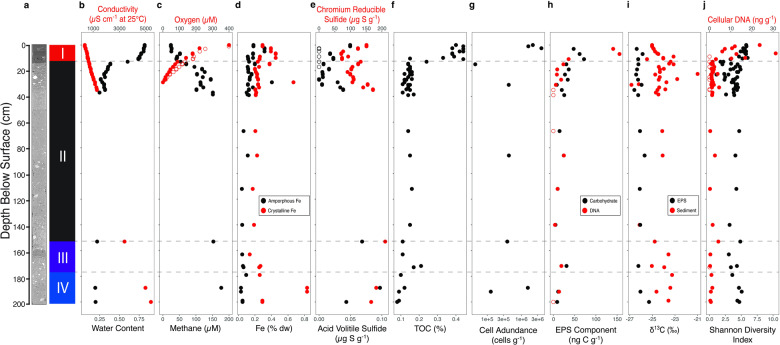
Composite depth profile of analytes from SLM sediment cores. Physical (**a**, **b**), geochemical (**c**–**f**), isotopic (**i**), and microbiological (**g**, **h**, and **j**) data are plotted. The Y-axis represents the composite sediment depth, as described in the Supplementary Information, and the dashed horizontal lines are the boundaries between the stratographic units. The open and closed circles for oxygen (**c**) represent two separate cores used for measurements, while the open circles for sulfide (**e**), EPS component (**h**), and cellular DNA (**j**) represent values too low to precisely measure and are plotted at the procedural detection limit. The oxygen concentration data are from Michaud et al. [47]; TOC and δ^13^C values are from Venturelli et al. [16].

Total organic carbon (%TOC) and bulk stable carbon isotope composition (δ¹³C) for acid insoluble OM and EPS were determined with a Carlo-Erba NAN2500 Series-II Elemental Analyzer coupled to a continuous flow Thermo-Finnigan Delta+ XL isotope ratio mass spectrometer [16]. Sediment water content and measurement of porewater conductivity, sulfate, and nitrate followed the procedures used in studies of SLW (Supplementary Information; [6, 35]). Dissolved oxygen concentrations in sediment porewater were determined using a Clark-type microsensor which was calibrated using a 1M NaOH and 0.1M ascorbic acid solution for the 0% oxygen saturation standard and water bubbled with atmospheric air for 30 min as the 100% oxygen saturation standard. All calibrations were conducted at near in situ temperatures (3 °C). Methane concentrations were measured by gas chromatography as previously described [7]. To enable inclusion of sampling depths in correlative analyses (e.g., RDA plot) for which methane data are not available (i.e., nine of the depths sampled in units II to IV), the values were interpolated from two linear regression models fit to the data: one using 19 sample depths between 0 and 35 cm and the other with 3 samples from depths of 37, 153, and 189 cm (Fig. 2c). Highly reactive nanoparticle iron was extracted from sediments using a two-step chemical leach, an ascorbate solution, and a subsequent dithionite leach (Supplementary Information; [36–39]). Solid phase sulfides were distilled from the sediment with a standard two-step sulfide extraction [40], resulting in acid volatile sulfide (AVS; 6 M HCl) and chromium reducible sulfide (CRS; 2M CrCl_2_ with 2M HCl) fractions based on the solutions used for separation. Sulfide trapped as ZnS during distillation was quantified spectrophotometrically using the diamine reaction [41]. Sulfate reduction was measured using the cold chromium distillation method (Supplementary Information; [42, 43]).

### Molecular biological analysis of the microbial communities

Genomic DNA was extracted from thawed samples using the same extraction method as sediments from SLW [6, 8] and the WGZ (Supplementary Information; [20]). The V4 region of the 16S rRNA gene was amplified using primers from Caporaso et al. [44] and sequenced on an Illumnia MiSeq platform using the 2 × 300 v3 run format. R version 4.1.1 with the DADA2 v1.16.0 package was used to process reads, assigning taxonomic value based on the SILVA database v138 training set (Supplementary Information; [45]). The sequencing data are available in the Sequence Read Archive of NCBI under the projects PRJNA790995 (SLM data), PRJNA244335 (SLW data), and PRJNA869494 (WGZ data).

## Results

### Downcore gradient of biogeochemical variables and microbial cells

Sedimentologic analysis (Supplementary Information) revealed four lithofacies in the 2.06 m SLM sediment profile: unit I (0–11.5 cm), unit II (11.5–147 cm), unit III (147–177 cm), and unit IV (177–206 cm; Fig. 2a). All data collected and analyzed from the profile are provided in Supplementary Data File 1. Specific conductivity of the sediment pore water was lowest in the surface (360 μS cm^−1^) and increased linearly with depth (Fig. 1b). Extrapolation of the conductivity profile using linear regression implies a seawater composition endmember at a sediment depth of 25–28 m. Oxygen decreased to below detectable levels (<0.4 µM) in sediments deeper than 30 cm (Fig. 2c); nitrate ranged from 2.2 to 4.0 μM in pore waters near the surface and decreased to below the level of quantification (2.2 µM) at depths >20 cm; and sulfate increased linearly from 0.3 mM in surficial samples to 0.8 mM at depths of 35 cm (Supplementary Data File 1). Nanophase crystalline iron (assumed primarily aged ferrihydrite, goethite, and hematite) exceeded (124 to 255% higher) that of highly reactive amorphous Fe (oxyhydr)oxides (Fig. 2d), and their concentrations are positively correlated (*r*_*s*_ = 0.41, *n* = 56, *p* < 0.005). The amorphous iron concentration at anoxic depths (average of 0.089%) is ~twofold lower on average than that at oxic depths (0.18%), with an exception being 189 cm in unit IV (Fig. 2d), which contained the highest crystalline iron (0.83%) and lowest amorphous iron (0.031%) concentrations observed in the profile.

There was a steep decreasing gradient in TOC concentration with depth between unit I and II (Fig. 2f and Supplementary Information), and based on a linear regression of the log-transformed data, a power law relationship describes most of the TOC concentration variation in the profile (m = −1.94, r^2^ = 0.58). Dissolved pore water methane concentrations in unit I increased linearly with depth from 24 µM at the surface to 151 µM at 37 cm (Fig. 2c). Methane concentration was negatively and significantly correlated to the concentration of oxygen (*r*_*s*_ = −0.88, n = 37, *p* < 0.001) and TOC (*r*_*s*_ = −0.71, *n* = 46, *p* < 0.001). Chromium reducible sulfide (CRS; e.g., FeS_2_) and oxygen are weakly negatively correlated (*r*_*s*_ = −0.25, *n* = 37, *p* > 0.05). Although the concentration of CRS exceeded that of AVS by ~5000-fold on average (Fig. 2f), the profile for both analytes with depth followed a similar trend (Supplementary Information).

No fluorescent dissolved OM was detectable in samples of the extracted EPS and total protein concentration in the samples was below the procedural level of detection (58 ng g^−1^). Similar amounts of carbohydrate (5–72 ng C g^−1^) and DNA (EPS-DNA; 3.8–156 ng C g^−1^, 4 samples were <3 ng g^−1^) were associated with the EPS (Fig. 2h), and their concentrations are significantly positively correlated (*r*_*s*_ = 0.87, *n* = 17, *p* < 0.001). Based on maximum values for total carbon in EPS from the surficial sediments (22 μg C g^−1^, Supplementary Information), carbohydrate and DNA are estimated to represent 2.7–14.2% of the EPS-carbon. Near Edge X-ray Absorption Fine Structure (NEXAFS) spectra of the EPS displayed main peaks representing aromatic (~285 eV; C = C), aliphatic (~287.5–288 eV; C-H) and carboxylic (~288.5 eV; R-COOH) functional group transitions, which is likely indicative of degraded organic material (i.e., proteins and amino acids) of microbial origin (Supplementary Information). The δ^13^C values of EPS are relatively constant with depth and range from −26.6‰ (35 cm) to −25.7‰ (86 cm; Fig. 2i), consistent with a marine source of the OM (Supplementary Information; [16]). In samples from the overlying water column, material that passed through a 0.2 μm pore-size filter had the lowest δ^13^C value observed (−30.0‰).

Direct counts of cells extracted from the sediments showed that their abundances were highest at unit I depths of 0 to 4 cm (2.6 ± 0.10 × 10^6^ cells g^−1^; ±SEM; Fig. 2g). Unit II and III depths sampled between 30 and 153 cm had similar cell concentrations (4.1 ± 1.0 × 10^5^ cells g^−1^), whereas there is ~tenfold range for these values in unit IV (1.6 ± 0.05 × 10^6^ to 1.3 ± 0.13 × 10^5^ cells g^−1^, 189 and 192 cm, respectively). Sediment DNA biomass shows a similar trend as cell abundance, is positively correlated to oxygen (*r*_*s*_ = 0.71, *n* = 30, *p* < 0.001) and TOC (*r*_*s*_ = 0.50, *n* = 48, *p* < 0.001) concentrations, and is negatively correlated to methane concentrations (*r*_*s*_ = −0.59, *n* = 45, *p* < 0.001). Assuming an average DNA content for marine bacteria of 2.5 fg of DNA cell^−1^ [46], the efficiency of the cell extraction procedure is inferred to be >20% except for the sample from 15 cm (7%), where the lowest cell concentration was observed (Fig. 2g).

### Microbial community composition, structure, and diversity in SLM sediments

The vast majority of ASVs from SLM sediment samples classify as Bacteria (97.3% of 5,605; see Supplementary Information) and 2.6% are archaeal (95 ASVs). Approximately 0.1% of the ASVs (<0.0004% of total reads) could not be classified at the domain level and 5.0% (3.7% of total reads) could not be classified at the phylum level. Species richness (Fig. S3) and diversity (Table 1 and Fig. S3b–d) were highest in unit I samples, and the Shannon diversity index significantly negatively correlates to depth (*r*_*s*_ = −0.31, *n* = 58, *p* < 0.05; Fig. 2j). The trend of decreasing downcore diversity is consistent with maximum Chao1 richness estimates of 787 ± 322 (Table 1) in the surficial sediments that decrease with depth and indicate that, on average, species richness in unit I was approximately fourfold higher than in unit II and nearly twofold higher than that observed in units III and IV.

**Table 1:** Summary of sequencing results and diversity estimation metrics.

	Unit I	Unit II	Unit III	Unit IV
Depth (cm)	0–11.5	11.5–147	147–177	177–203
Number of samples	12	37	4	5
Number of total reads/Reads per sample	3,864,908/322,076 ± 111,640	6,047,561/163,448 ± 104,015	748,867/187,217 ± 96,097	1,049,518/209,904 ± 14,367
Total ASVs	3218	2453	1206	1331
ASVs > 0.1% of total	199	171	133	133
Shannon diversity index	5.46 ± 0.41	3.59 ± 0.94	3.88 ± 0.84	4.46 ± 0.55
Chao1	787 ± 322	183 ± 173	458 ± 334	518 ± 184
Inverse Simpson Index	110 ± 45	25 ± 16	21 ± 13	39 ± 20
Simpson Diversity Index	0.99 ± 0.01	0.92 ± .09	0.93 ± .06	0.96 ± .03

±standard deviation.

Pairwise comparison analysis was used to identify ASVs with relative abundances that significantly correlate to physical, biogeochemical, and microbiological variables in the composite sediment profile (Table 2). This revealed significant correlations to physical properties (depth, conductivity, or water content) or potential electron donors (TOC, methane, CRS, and AVS) and acceptors [oxygen and amorphous Fe] available in the sediments. Specifically, 15% of the taxa are positively correlated to the TOC concentration (19 ASVs are strongly significantly correlated) and 6 to 8% of taxa are positively correlated to the concentration of CRS, AVS, or methane (Table 2). A substantial fraction of the ASVs are positively correlated to the potential electron acceptors oxygen (16% of the total) and Fe (10 and 16% for amorphous and crystalline Fe, respectively). There are also taxa with relative abundances that correlate to physical properties (depth, pore water conductivity, and water content) or microbiological variables (e.g., Shannon Diversity Index and cell abundance; Table 2) that, in general, tended to monotonically increase or decrease with sediment depth (Fig. 2).

**Table 2:** Summary statistics from pairwise comparison of ASV relative abundance to environmental variables and Pearson correlation coefficients. A statistically significant result has a p value ≤ 0.05.

Parameter	Positively correlated		Negatively correlated	
	Strong	Moderate	Strong	Moderate
	0.6 ≤ |r| <1.0	0.2 ≤ |r| <0.6	0.6 ≤ |r| <1.0	0.2 ≤ |r| <0.6
	ASV/%^a^	ASV/%^a^	ASV/%^a^	ASV/%^a^
Oxygen	44/0.7%	851/15.2%	5/0.09%	473/8.4%
Amorphous Fe	0/0%	545/9.7%	7/0.1%	477/8.5%
Crystalline Fe	0/0%	884/16%	0/0%	75/1.3%
CRS	0/0%	312/5.6%	0/0%	489/8.7%
AVS	0/0%	383/6.8%	0/0%	7/0.1%
Methane	3/0.05%	456/8.1%	40/0.7%	832/15%
TOC	19/0.3%	847/15%	0/0%	305/5.4%
Sediment *δ*^13^C	0/0%	5/0.09%	0/0%	280/5.0%
Depth	21/0.4%	547/9.8%	43/0.8%	905/16%
Conductivity	0/0%	338/6.0%	137/2.4%	800/14%
Water Content	123/2.2%	823/15%	0/0%	330/5.9%
DNA	29/0.5%	1145/20%	0/0%	9/0.2%
Shannon Diversity Index	152/2.7%	1221/22%	0/0%	5/0.09%
EPS-Carbohydrate	5/0.09%	84/1.5%	63/1.1%	120/2.1%
EPS-DNA	19/0.3%	186/3.3%	2/0.03%	35/0.6%
EPS *δ*^13^C	55/1.0%	137/2.4%	1/0.02%	4/0.07%
Cell Abundance	278/5.0%	0/0%	0/0%	0/0%

^a^The percentage relative to the total number of ASVs.

**Fig. 3:**
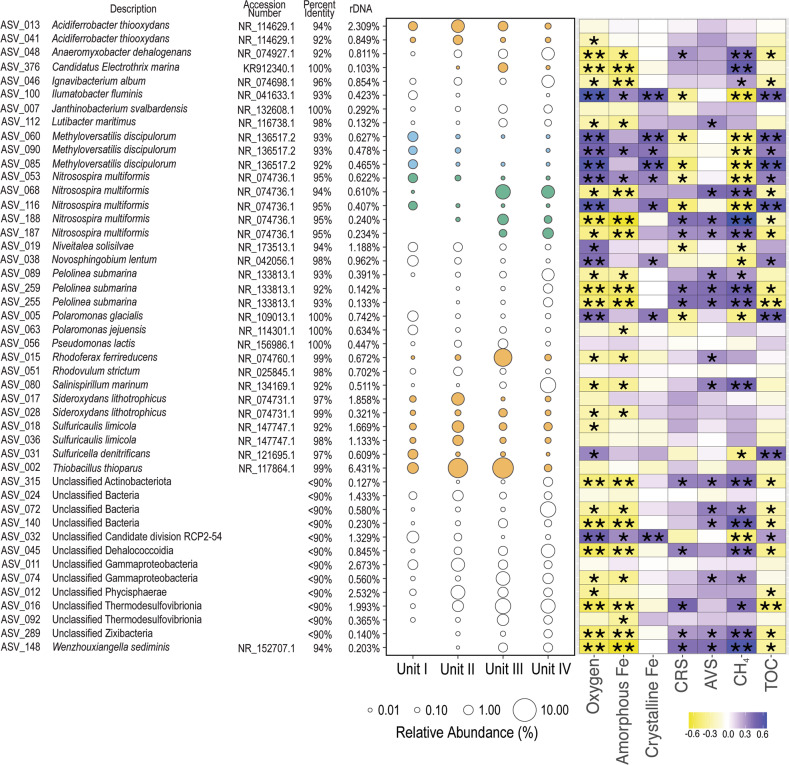
Bacterial and archaeal ASVs with a relative abundance >1% of total sequences in at least one of the four sediment units (I to IV) from SLM. ASVs are listed in alphabetical order and with the percent 16S rRNA gene identity to their nearest taxonomic neighbor and its GenBank accession number. Bubble color indicates ASVs having a nearest neighbor with the capacity to use reduced iron and sulfur compounds (orange), ammonia (green), or C1 compounds (blue) as electron donors. Heat map colors represent the correlation coefficient for the relative abundance of each ASV to environmental variables using Spearman’s correlation test. ** = *p* ≤ .001; * = 0.05 ≥ *p* > .001.

The most abundant ASV in the SLM sediment profile (ASV_2, 6.5% of the total reads) is closely related to the 16S rRNA gene sequence of *Thiobacillus thioparus*, has the highest relative abundance in units II and III (9.5 and 11%, respectively, of the total reads), and does not correlate to any of the physical, chemical, or microbiological variables (Fig. 3). Based on their nearest neighbors (i.e., *Acidiferrobacter*, *Sideroxydans*, *Sulfuricaulis*, *Sulfuricella*, and *Candidatus* Electrothrix), there may be at least eight other abundant taxa in the profile capable of utilizing reduced sulfur and/or iron compounds as electron donors. In fact, the relative abundances for some of these ASVs [i.e., ASV_28 (*Sideroxydans*) and _376 (*Candidatus* Electrothrix)] correlate to the concentration of amorphous Fe (Fig. 3). Five ASVs are most closely related to *Nitrosospira multiformis*, albeit distantly (94 to 95% 16S rRNA gene identity), and if these are species capable of ammonia-oxidization, then their pattern of distribution in the sediment profile suggests considerable ecophysiological differences among the taxa. For example, ASV_53 and ASV_116 are significantly positively correlated to the oxygen and TOC concentration, whereas ASV_68, ASV_188, and ASV_187 are significantly negatively correlated to these variables (Fig. 3). Three ASVs (ASV_60, _85, and _90) with high relative abundances in unit I significantly and positively correlate to the oxygen and TOC concentration, and significantly and negatively correlate to the concentration of methane. Although these ASVs are distantly related to the methylotrophic species *Methyloversatilis discipulorum* (≤93% 16S rRNA gene identity), such a pattern of distribution is consistent with that for an aerobic C1 metabolizer.

**Fig. 4:**
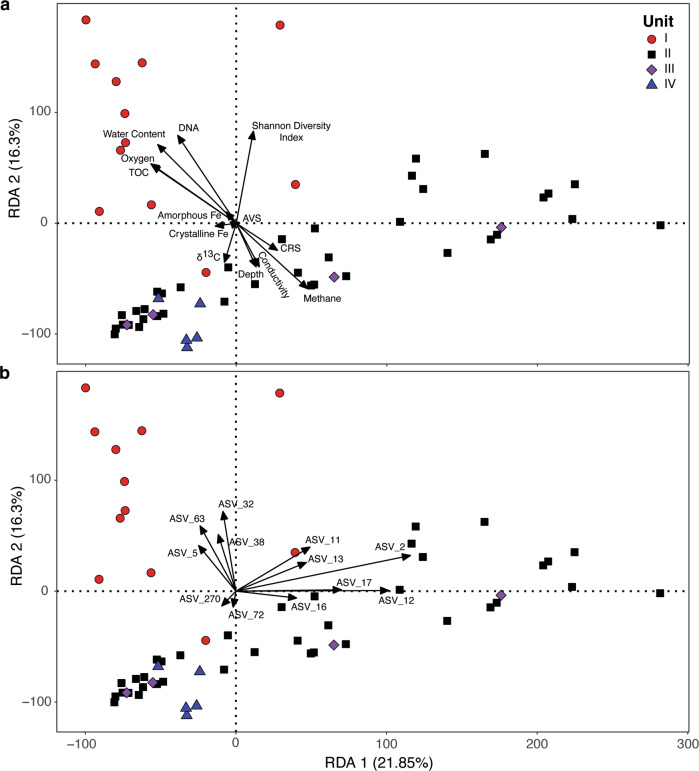
Redundancy analysis of microbial assemblages in the SLM sediment profile. The first two dimensions of the ordination plot are shown for (**a**) environmental parameters and (**b**) ASVs that are explanatory variables of community structure (black arrows). The classification of ASVs is shown in Fig. 3 with the exception of ASV_270, which had a relative abundance of 0.15% of total sequences and classifies within the genus *Pseudomonas*. The two axes in the RDA model explain 38.15% of the cumulative variance in the community composition. Based on a permutation test of total variance for environmental variables and community composition (*p* value ≤0.001; ANOVA, 999 permutations), the dispersion of unit I in ordination space is significantly different from units II–IV.

Redundancy analysis (RDA) was used to test the hypothesis that key biogeochemical variables and taxa correlate to community composition (Fig. 4). Unit I samples form a loose cluster that is significantly different (*p* value <0.001; Adonis) from the underlying units, but there is not a significant difference among samples from units II-IV (Fig. 4). Not only are samples from units II and III different from unit I, but they are also relatively heterogeneous within themselves. Nonetheless, fourteen samples (predominantly from unit II) are clearly separated from the other samples along the first RDA axis (i.e., far right of plots in Fig. 4), do not share common ordination space with depths from the other units, and have community compositions that do not significantly correlate to explanatory variables (Fig. 4a). Based on a Mantel test using Spearman’s rank correlations and Euclidean distance, community composition is significantly positively correlated to TOC (*r*_*m*_ = 0.17, *p* < 0.05), water content (*r*_*m*_ = 0.17, *p* < 0.05), methane (*r*_*m*_ = 0.19, *p* < 0.001), and the Shannon Diversity Index (*r*_*m*_ = 0.50, *p* < 0.001). The relative abundance of the six most abundant ASVs in the samples (ASV_2, _11, _12, _13, _16, and _17; Fig. 3) are correlated to community composition along RDA 1 axis (Fig. 4b), while the unit I samples are positively correlated to four ASVs (ASV_5, _32, _38, and _63; Fig. 4b) that are the most abundant taxa at these depths (Fig. 3).

### Similarity in sediment communities and populations of the Siple-Gould Coast region

**Fig. 5:**
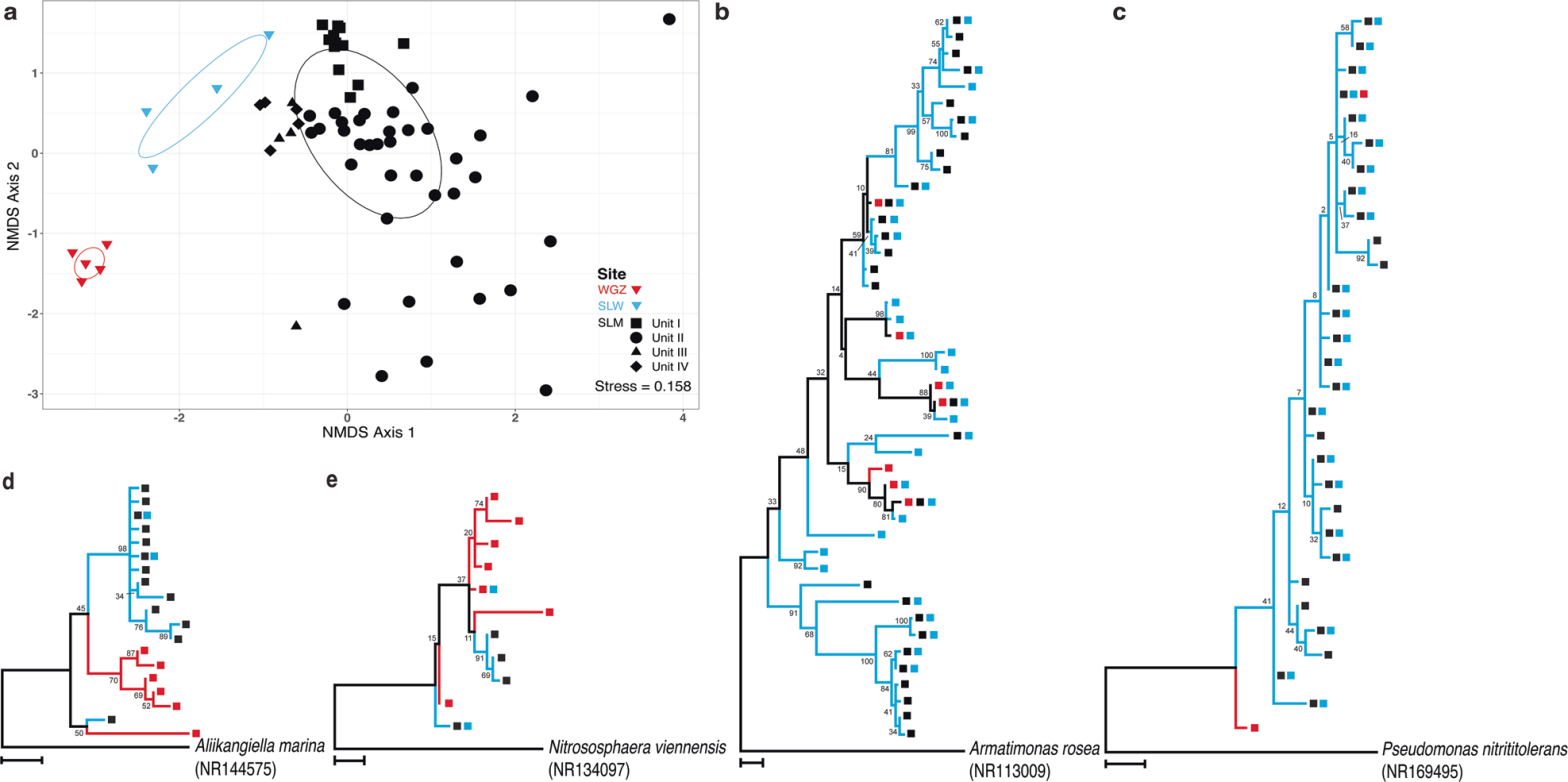
Comparison of communities and taxa in sediment samples from SLM, SLW, and the marine WGZ. **a** Nonmetric multidimensional scaling (NMDS) plot of samples from the communities. Distance was calculated using Bray-Curtis dissimilarly for ordination and Adonis was used for testing statistical significance. Sample depths (at 2 cm intervals) for WGZ are between 0 and 10 cm [20], and for SLW, the median depth is 1, 5, 19, and 35 cm [8]. The SLM unit sample depths are shown in Fig. 2. Ellipses are 50% confidence intervals. **b**–**e** Maximum likelihood analysis based on aligned partial 16S rRNA gene sequences from related ASVs with >94% identity to each other. Colored lines highlight lineages found exclusively in marine (red; WGZ samples) versus subglacial (blue; SLW and SLM samples) sediments. The colored squares for each taxon represent the site of origin for the ASV: SLM (black), SLW (blue), and WGZ (red). Phylogenetic analysis was carried out with taxa affiliated with the class b) *Anaerolineaceae* and genera c) *Candidatus* Nitrotoga, **d** *Pseudohongiella*, and (**e**) *Candidatus* Nitrosopumilus. The scale bar represents 0.02 fixed substitutions per nucleotide position. Bootstrap values are shown at the nodes and represent 100 replications.

Sediment samples from the two WAIS subglacial lakes (SLM and SLW) and the marine WGZ contain significantly different communities (*p* value < 0.001; Adonis, Fig. 5a). Samples from unit I of SLM occupy a similar region of NMDS ordination space as the most surficial sediment sample from SLW (0 to 2 cm), but their communities tend to become more dissimilar with depth in unit I. However, the assemblages in units III and IV are more similar to those in the diamict sampled from SLW than they are to unit I. In contrast to the trend observed with depth in SLM, the deepest SLW community samples are closer in ordination space to surficial sediments from the WGZ (Fig. 5a).

SLM sediment communities share the most ASVs (715) with SLW (Fig. S4), which represent 46% of SLW’s ASVs and 75% of its total sequencing reads. Fewer taxa are shared with the WGZ (62 ASVs), with 80% of these originating from unit III. Of the ASVs common to sediments at all three sites (32 ASVs; Table S2), SLM units II and III contain marginally more shared ASVs (28 of 32) than units I and IV (23 and 26, respectively). In addition to sharing abundant taxa with identical 16S rRNA gene sequences, a diversity of phylogenetically related sequence types (>94% identity) are observed among the sites (examples in Fig. 5b–e). Moreover, maximum likelihood analysis showed that phylotypes detected in the freshwater sediments of SLM and SLW tend to cluster together. Clades containing ASVs found only in sediments from the marine WGZ generally excluded taxa that were observed in SLM and SLW (e.g., Fig. 5d, e).

## Discussion

The relative abundance of prevalent taxa in the SLM sediment profile (Fig. 3 and Table 2) and structure of the communities (Fig. 4) correlate to changes in key environmental parameters with depth, implicating specific bacteria and archaea in biogeochemical reactions that are linked between the surficial sediments and water column. When the lake sediments of unit I are compared to those in the underlying diamict (units II to IV), there is an increase in heterogeneity of composition with depth across units, and fewer environmental variables correlate well to the structure of these diamict communities (Fig. 4a). Although variation of species distributions is expected over gradients of oxygen and OM (Fig. 2), shifts in microbial composition with depth could also reflect changes in depositional history. Given the low microbial activities in SLM’s aerobic sediments [47], its species may be interspersed with those originating from material exchange with the oceanographic system and upstream components of the subglacial hydrological system. As such, portions of the assemblages we have documented predate the lake [48], and in the deepest units, their origin could even precede the Middle Holocene marine incursion [18]. Comparison of SLM’s unit I to the underlying diamict thus offers an opportunity to differentiate microbial populations of the subglacial lake ecosystem from those that may be more representative of sediments associated with grounded ice conditions.

Laminated sediments are resolvable in the upper 12 cm of surficial sediments obtained from SLM (Fig. 2a), and these high water content sediments are interpreted as a ~180 year deposition record since the lake was formed [48]. Similar to TOC measurements at SLW (0.3–0.5%; [7]), the sediment in unit I contains low TOC (~0.4%) in comparison to Siberian glacial lakes (~1.6%; [49]) and alpine lakes (~0.8%; [50]), with TOC depleted to values <0.2% at the oxic-anoxic transition zone (Fig. 2f). A subglacial lacustrine origin for unit I is consistent with the changes in microbial cell concentration (Fig. 2g), composition (Fig. 3), richness (Table 1), diversity (Fig. 2j), and community structure (Fig. 4) observed across the unit I:II boundary. Two-thirds of the taxa in unit I are not observed in the deeper units, nor are they abundant in samples of the overlying water column (5% are shared; [51]) or ice (2% are shared with taxa in ice melted for the borehole; [51]). Nearly half (46%) of the ASVs in the sediment samples from SLW [8] were also identified in SLM, with the greatest percentage of these shared with those in unit I (68%), accounting for their similar assemblage structures (Fig. 5). These relationships indicate that the hydrological pathways beneath WAIS access similar sediments in the hydrological basins where SLM and SLW are located. It is also notable that the SLW sediment assemblages similar to those in unit I of SLM inhabit a unit II-type sediment (i.e., diamict) with contrasting geochemical conditions, implying that both dispersal and geochemical conditions are contributing drivers of community structure.

Autochthonous production of OM in the water column is a possible explanation for the higher TOC concentrations in samples from unit I (Fig. 2f). However, at the dark CO_2_ fixation rate measured in a sample from the sediment: water interface (0.056 ± 0.010 nmol C L^−1^ d^−1^) and neglecting remineralization, accumulation estimates over SLM’s ~180 year history are ~30,000 times less than the excess TOC observed in unit I. Even if this rate is applied to the entire water column, the mismatch is 300-fold. Communities in the sediment represent another potential source of autochthonous OM, which is a contention supported by the abundant putatively chemoautotrophic taxa identified in the sediment samples (Fig. 3). These include ammonia oxidizing bacteria (*Nitrosomonas, Nitrosospira*, MND1, and GOUTA6) and archaea (*Candidatus* Nitrosopumilus and *Candidatus* Nitrosoarchaeum), together with nitrite oxidizing bacteria (*Candidatus* Nitrotoga and *Nitrospira*). Bacteria in iron- and/or sulfide-oxidizing genera are also abundant (*Thiobacillus, Sulfurifustis*, *Sideroxydans*, and *Gallionella*; Fig. 3), including phylotypes closely related to those documented in subglacial sediments from WAIS [8, 52] and alpine glaciers [53], as well as a lake in the McMurdo Dry Valleys, East Antarctica [54]. Nevertheless, the stable isotopic composition of the TOC and relatively uniform values of δ^13^C for EPS with depth (Fig. 2i) are consistent with marine-derived OM serving as the major carbon assimilation source to SLM’s sediment community. It is possible that low rates of autochthonous production in SLM are responsible for reliance of the communities on legacy marine OM. The ^14^C-bearing OM has been preferentially remineralized compared to ^14^C-free OM in the sediments [16], implying the Holocene-aged marine OM is more bioavailable relative to the more ancient kerogenic carbon stocks (e.g., Oligocene and Miocene age) stored beneath WAIS.

Despite methane concentrations as high as 176 μM in the depth profile (Fig. 2c), no methanogenic archaea were identified in SLM sediments, and they were extremely rare in sediment samples from SLW [8]. However, it is important to note that our approach is optimized for detection of bacterial and not archaeal diversity. In comparison, abundant taxa related to methane oxidizing bacteria and archaea are observed where large changes in methane concentration occur in the sediment (Fig. 2c), having their highest relative abundances in samples near the surface and oxic-anoxic transition (~30 cm; Fig. 3). The ASVs classifying within the genus *Methylobacter* are identical to those documented in SLW’s water column and sediments [7, 8] and phylogenetically related to those reported in subglacial outflows from the Greenland Ice Sheet [55]. Collectively, these observations confirm a microbial sink for methane in the surficial sediments, indicate SLM contains methane-oxidizing taxa phylogenetically unresolvable from those observed at SLW (based on 16S rRNA gene comparison), and suggest a biological methane source that originates from sediment depths >2 m.

Based on the dissolved oxygen concentration profile (Fig. 2c), >90% of the oxygen consumption (0.6 mmol O_2_ m^−2^ d^−1^; [47]) occurs in the upper 5 cm of the sediments. Given the lower nitrate concentrations at sediment depths near the oxic-anoxic transition zone (~30 cm; Supplementary Data File 1) relative to the surface sediments, anaerobic respiration of nitrate has likely occurred. Indeed, a close phylogenetic relative of the obligate chemolithoautotroph *Thiobacillus thioparus*, which couples the oxidization of reduced sulfur compounds to dissimilatory nitrate reduction [56], is the most abundant ASV observed in the sediment profile (Fig. 3; ASV_2). At anoxic depths where nitrate concentrations are low, the reactive Fe concentrations are lower than values observed at surficial depths (Fig. 2d), implying that it may be used as an electron acceptor. This is consistent with the distribution of ASVs related to the facultative iron reducing genus *Rhodoferax*, which are at their highest abundances in units III and IV (e.g., ASV_15) and are negatively correlated to Fe concentrations (Fig. 3). Despite representation of *Desulfosporosinus* and other known sulfate reducing bacteria (0.5% of the sequences in unit IV), sulfate reduction activity was below detection in the SLM sediments. It is possible that conditions in the past were more reducing and supported sulfate-reduction in the sediments. Alternatively, they could represent dormant but persisting marine sedimentary taxa that were deposited when the region transitioned into an ice shelf environment during the Middle Holocene [16].

WAIS governs direct exchange between marine and subglacial ecosystems, effectively controlling distribution patterns and gene flow to populations in SLM. The marine incursion during the Holocene [16] could have resulted in marine taxa being deposited in sediments upstream of SLM. Hence, identical taxa shared among samples from SLM, SLW, and the WGZ site [Fig. S4; e.g., *Thiobacillus* (ASV_2), *Sulfurifustis* (ASV_18), and *Nitrotoga* (ASV_190)] could have a marine source. Conversely, these ASVs may have originated from a subglacial biome that exists in the freshwater hydrological system beneath WAIS. The latter possibility, together with the isolated nature of the contemporary subglacial environment, raises fundamental questions about the origin of subglacial microbial populations, their evolutionary relationships to species in sediments from other subglacial regions, and the scale of the microbial reservoir beneath the ice sheet. Microbial concentrations in sediment units II to IV ranged from 0.04 to 1.5 × 10^6^ cells g^−1^, and assuming a subglacial aquifer depth of 1 km [57] and sediment density of 2 g cm^−3^, we estimate that sediments under the Antarctic ice sheet (1.0 × 10^7^ km^2^; [58]) contain from 0.08 to 3 × 10^28^ cells. While these values are 10- to 500-fold lower than prior estimates [58], they nevertheless imply that the number of microbes in Antarctica’s subglacial aquifer may exceed that for all freshwater ecosystems globally by more than an order of magnitude [58, 59].

We found that the modern microbial sediment community of SLM (i.e., unit I) has a structure and composition similar to that in SLW (Fig. 5). Nearly half of ASVs (46%) identified in SLW sediments were also present in SLM (Fig. S4), and closely related taxa observed in the lakes are phylogenetically distinct from those found in marine sediments at the WGZ (Fig. 5b–e). Based on these results, we hypothesize that the distribution and evolutionary relationships of microorganisms in SLM and SLW are linked via dispersal through the subglacial hydrological system beneath WAIS. Testing this hypothesis will require implementing molecular evolutionary and population genetic approaches that target loci with phylogenetic resolutions superior to the 16S rRNA gene. If microbes in the SLM and SLW ecosystems originated from upglacial sources that inoculated the lakes, then it is possible these populations could have been isolated beneath Antarctic ice for millions of years [19, 60]. Accordingly, the sediment community of SLM may be one component of an extensive subglacial metacommunity beneath WAIS that is hydrologically, biogeochemically, and evolutionary linked through ice sheet behavior and the subglacial transport of microbes, water, and sediments.

## Supplementary Information

Supplementary Information

Supplemental Data File 1

## Data Availability

The sequencing data are available in the Sequence Read Archive of NCBI under the projects PRJNA790995 (SLM data), PRJNA244335 (SLW data), and PRJNA869494 (WGZ data).
