# Supplementary material for: Biogeochemical and historical drivers of microbial community composition and structure in sediments from Mercer Subglacial Lake, West Antarctica
Source: ISME Commun. 2023 Jan 30;3:8. doi: 10.1038/s43705-023-00216-w (PMC9886901; doi:10.1038/s43705-023-00216-w)
Supplement: Supplementary file 1 — Supplementary Information [file 43705_2023_216_MOESM1_ESM.docx]

**Supplementary Information for the research article: Biogeochemical and historical drivers of microbial community composition and structure in sediments from Mercer Subglacial Lake, West Antarctica**

Christina L. Davis^1^, Ryan A. Venturelli^2^, Alexander B. Michaud^3^, Jon R. Hawkings^4^, Amanda M. Achberger^5^, Trista J. Vick-Majors^6^, Brad E. Rosenheim^7^, John E. Dore^8^, August Steigmeyer^9^, Mark L. Skidmore^9^, Joel D. Barker^10^, Liane G. Benning^11,12^, Matthew R. Siegfried^13^, John C. Priscu^14^, Brent C. Christner^1^*, and the SALSA Science Team†

^1^Department of Microbiology and Cell Science, University of Florida, Gainesville, FL, USA

^2^Department of Geology and Geological Engineering, Colorado School of Mines, Golden, CO, USA

^3^Center for Geomicrobiology, Aarhus University, Aarhus, DK and present address: Bigelow Laboratory for Ocean Sciences, East Boothbay, ME, USA

^4^Department of Earth and Environmental Science, University of Pennsylvania, Philadelphia, PA, USA

^5^Department of Oceanography, Texas A&M University, College Station, TX, USA

^6^Department of Biological Sciences, Michigan Technological University, Houghton, MI, USA

^7^College of Marine Sciences, University of South Florida, St. Petersburg, FL, USA

^8^Department of Land Resources and Environmental Sciences, Montana State University, Bozeman, MT, USA

^9^Department of Earth Sciences, Montana State University, Bozeman, MT, USA

^10^School of Earth and Environmental Sciences, University of Minnesota, Minneapolis, MN, USA

^11^GFZ German Research Centre for Geosciences, Telegrafenberg, Potsdam, Germany

^12^Department of Earth Sciences, Freie Universität Berlin, Berlin, Germany

^13^Department of Geophysics, Colorado School of Mines, Golden, CO, USA

^14^Polar Oceans Research Group, Sheridan, MT, USA

†Full list provided at end of this document

*Author to whom correspondence should be addressed: [xner@ufl.edu](mailto:xner@ufl.edu)

**Supplementary Materials and Methods:**

*Site description*

SLM is located in the southern basin of Mercer Ice Stream (Fig. S1), has an average depth of 5 m, is part of an active subglacial hydrological system, and has well documented fill-drain cycles [1]. Based on 18 years of observational data, fill-drain cycles range from four to six years, and events in 2003 and 2017 were triggered by drainage of the lake immediately upstream of SLM’s hydrological flow path (Conway Subglacial Lake; [2]). In mid-2018, SLM began to rapidly drain and remained in a drainage phase throughout the period of field operations and sampling [2].

The site selected for drilling into SLM (84.640287 S, 149.501340 W) was near the center of the lake where the largest ice sheet surface height anomalies were observed (Fig. S1), which corresponds to changes in the thickness of the subglacial water column [3]. On 26 December 2018, a 1087 m deep borehole of ~0.4 m diameter through the ice was completed into SLM using environmentally clean hot water drilling [4-7] and deployment procedures [8]. During the time of subglacial access (26 December 2018 to 5 January 2019), SLM’s water column observed depth was 15 m. The drilling and sampling of sediments at SLW (84.240 S, 153.694 W) and the marine WGZ (84.3354 S, 163.6119 W) were performed as part of the Whillans Ice Stream Subglacial Access Research Drilling (WISSARD) project and occurred during the 2012-2013 and 2014-2015, respectively, austral field seasons.

*Sampling SLM’s water column and sediments*

A large volume water filtration system (WTS-LV; McClane Inc.) was deployed to sample microbial cells and particulates in the lake water column [4-6]. A sediment catcher constructed out of polyethylene was affixed at the base of the WTS-LV frame to facilitate the collection of bulk surface (BS) sediments during each cast. The WTS-LV was carefully lowered through the borehole into the lake until the winch load cell indicated unweighting of the sonde on the sediment surface, and the system was then raised into the middle of the water column (7.5 m above the water-sediment interface) for sampling. Particulates in 15 to 18 L of water were sequentially concentrated on 142 mm Supor PES membrane filters (Pall Corp.) with pore sizes of 3.0, 0.8 and 0.2 µm. After the WTS-LV was recovered, returned to the field laboratory, and the filter housing was disassembled, the water retained between each filter stack was collected in sterilized 125 mL polypropylene Nalgene bottles. BS samples recovered by the WTS-LV sediment catcher were aseptically transferred into sterile Whirl-Pak bags. All samples were stored at -20ºC during transport to the University of Florida and until they were thawed for processing.

An Uwitec mulitcorer customized for borehole deployment was used to obtain surficial sediment cores from SLM [9]. Three multicores that ranged in length from 37 to 43 cm were sampled for microbiological analysis. A borehole gravity coring device [9] was used to retrieve two cores with a length of 1.00 and 1.76 m. Information for the individual cores samples, as well as the BS sediments, analyzed in this study is detailed in Table S1. The sample designations are based on the collection instrument, cast, and individual cores recovered during a cast. For example, MC1B is the second core (B) recovered from the first multicore cast (MC1); 01FF is the first freefall core; and BS3 is bulk sediment that was collected from the third WTS-LV cast.

Multicores MC1B and MC1C were collected from the same cast on 31 December 2018 and MC4C was collected from a cast on 5 January 2019. Based on the flow rate for Mercer Ice Stream (~0.64 m d^-1^; [9]), the estimated distance between the two coring sites on the lakebed was approximately 3 m. Importantly, ice stream movement allowed the coring of different sites on the lakebed with each deployment and reduced sediment fallout from the region where the borehole was melted [9]. Sediment was collected at discrete depths in the cores as previously described [10-12], but the three multicores were not sampled simultaneously. MC1B (38 cm) was extruded <24 h after collection and sampled at 2 cm intervals. MC1C (43 cm) was stored at 4ºC, shipped directly to the Oregon State University Marine and Geology Repository (OSU-MGR; Corvallis, Oregon), and sampled on 1 May 2019 (~4 months after collection). At OSU-MGR, the core was sampled at a resolution of 4 cm and the material recovered was promptly frozen, shipped overnight to the University of Florida, and stored at -20ºC until processed. MC4C (37 cm) was stored at 4ºC for 2 weeks after collection before freezing at -20ºC, then it was transported to the University of Florida at -20ºC and stored frozen until it was thawed and sampled on 19 June 2019. The unconsolidated sediments in the upper ~10 cm of the MC4C core were collected as a slurry and 24 cm of the underlying diamict was sampled at 2 cm resolution. Bulk surficial sediments (BS3 and BS4) recovered on 1 and 4 January 2019 were collected at an approximate distance of 1 and 2.5 m, respectively, downstream from the MC1 core location [9].

The freefall gravity cores (01FF and 02FF) were collected at a distance of ~0.5 m from one another on the lakebed, and <2 m from the MC1 coring site [9]. The 100 cm 01FF core was sectioned <24 h after the core was recovered, two 5 cm full round core segments (from core depths of 45 to 50 cm and 80 to 85 cm) were sampled, and the remainder of the core was shipped to the OSU-MGR and stored at 4ºC. The 176 cm 02FF core was split into two sections after recovery, shipped directly to the OSU-MGR at 4ºC, and stored at this temperature for 4 months until sampling on 1 May 2019. Depth intervals of 5 cm were sampled from both freefall cores at OSU-MGR based on facies changes, computed tomography (CT)-scans, and obtaining an even distribution across the depth of the cores. All the sediment samples collected in the field and at OSU-MGR were frozen, shipped to the University of Florida, and stored at -20ºC until processed.

Whole-core CT-scans revealed stratigraphy across the sediment cores and guided the sampling of the split cores. The upper 11.5 cm of SLM sediments are composed of a laminated sequence [3] that is underlain by a stratigraphic sequence of three distinct units. The lithostratigraphic and sedimentological features identified in the lower SLM sediment units may be indicators for paleo-ice stream dynamics and hydrological basal conditions in this region of WAIS, while the laminated surficial unit represents a century-scale recorder of ice and lake history [3]. The following data from the MC1A, MC1C, 01FF, and 02FF cores were analyzed using Corewall Corelyzer software (https://csdco.umn.edu/resources/software/correlator): magnetic susceptibility and bulk elemental composition derived from a Geotek standard multi-sensor core logger and ITRAX X-ray fluorescence scanner, respectively. The horizontal patterns identified in the analysis were correlated to generate a composite depth scale that stratigraphically aligned depths from the different cores to allow comparisons over a 2.06 m profile [13].

*Enumeration of microbial cells in the sediment*

To enumerate DNA-containing cells in the sediments, frozen samples collected from discrete intervals (2 to 5 cm) intervals in the core samples were thawed and microbial cells associated with particles were extracted following established methods [14, 15]. Between 0.3 to 0.6 g of sediment (wet weight) was transferred to 15 mL Falcon tubes, followed by the addition of 6 mL of a filtered (0.2 μm pore size) and autoclaved solution of 3 mM NaCl (4 mL) and 2mL 1% w/v tetrasodium pyrophosphate (Na_4_P_2_O_7_). The slurries were shaken at 200 rpm for 1 h at 4 ºC. A 2 mL layer of 50% w/v Nycodenz (Thermo Fisher Scientific) was added to the bottom of the slurries with a 16 ga needle and the tubes were centrifuged for 1 h at 3000 x g. The uppermost aqueous layer was removed and stored at 4ºC. The Nycodenz layer was removed, and a second extraction was performed with 3 mM NaCl (4 mL) and 1% w/v Na_4_P_2_O_7_ (2 mL), followed by another layer of 2 mL 50% w/v Nycodenz. The top aqueous layer was again removed and stored separately from the first extraction at 4ºC, followed by removal of the Nycodenz layer. Remaining sediment was placed in a 65ºC oven for at least 4 days before measuring the dry weight. Both extractions were processed separately, and data were combined to calculate the number of cells per gram dry weight of sediment.

The cell extracts were filtered onto black 0.22 µm polycarbonate Isopore^TM^ filters (Millipore, Cat. No. GTTP04700) that had been pre-rinsed with 10 mL of Tris/Borate/EDTA buffer. Cells collected on the membranes were stained and enumerated as previously described [16]. After the addition of 1.5 mL of 25X SYBR^TM^ Gold (Life Technologies Corp., Cat. No. S-11494), the samples were incubated for 15 minutes in the dark before applying vacuum (<62 kPa). The filters were removed from the fritted glass support, mounted onto glass microscope slides using a 4 µL drop of antifade solution above and below the filter, and overlain with a coverslip. For each sample, the DNA-containing cells in 25 to 30 fields of view (area of 3.5 x 10^4^ µm^2^ per field of view) were enumerated using a Nikon ECLIPSE Ni epifluorescence microscope. The estimated number of cells per filter on the primary and secondary extractions were added and divided by the dry weight of the sediment sample to obtain the number of cells per gram dry weight of sediment. Procedural controls co-extracted with the samples were also processed and low cell numbers required observations from 60 fields of view for robust concentration estimates. The number of cells associated with the blanks was subtracted from the samples.

*Rates of dark carbon fixation*

Rates of dark carbon fixation at the sediment: water interface were determined following the procedure described in [10], with the same modifications. Briefly, quadruplicate incubations (4 live and 4 trichloroacetic acid-killed controls) of 10 mL each of water collected from the top of a sediment multicore tube (core MC1-B) were amended with a final concentration of ~1 μCi mL^-1^ of ^14^C-codium bicarbonate in the dark for 96.4 h at 4°C. Radioactivity and rates of dark carbon fixation were determined as described in [10].

*Extraction and analysis of Extracellular Polymeric Substances*

Extracellular polymeric substances (EPS) were extracted from sediment and water column samples using a protocol based on previously reported methods [17-19]. Thawed sediment (~8 g wet weight) was transferred to 50 mL conical tubes followed by the addition of 15 mL of 50 mM EDTA (pH 8.0). The samples were shaken at 300 rpm for 4 h at ~22 ^o^C, centrifuged at 4000 x g for 30 min, the supernatant was recovered, and a second extraction was performed on the residual sediment. The supernatants from the separate extractions were amalgamated, an equal volume of ice cold 99% v/v ethanol was added, and the samples were incubated at -20 ºC for approximately 48 hours to precipitate EPS. Samples were then centrifuged at 900 x g for 30 minutes and the supernatant was removed and discarded. The pelleted material was washed twice with ice cold 70% v/v ethanol, centrifuged at 900 x g for 10 min, and the residual ethanol was evaporated by air drying the samples for 12 h. Dried pellets of the material were used directly in subsequent chemical and isotopic analyses. Biochemical characterization of the EPS was conducted on aqueous samples that consisted of resuspensions of the pelleted material in 5 mL of autoclaved and filtered deionized water.

The carbohydrate component of the EPS was quantified using UV spectrophotometry [20]. One mL of the dissolved extract was added to 3 mL of concentrated sulfuric acid (18.4M) and the absorbance at 315 nm (A_315_) was measured using an Epoch^TM^ Microplate Spectrophotometer (Biotek Instuments) equipped with a 96 well plate reader. A linear regression of A_315_ data for standard glucose concentrations (10 to 100 mg L^-1^) were used to estimate the carbohydrate content in glucose equivalents. DNA that co-extracted with the EPS (EPS-DNA) was quantified using the Qubit™ dsDNA HS Assay Kit (Invitrogen). Quantification of protein in the extract was done using the Qubit™ Protein BR Assay Kit. The carbon content of the carbohydrate and DNA associated with the EPS was calculated assuming carbon was 40% and 41.8%, respectively, of the total sample mass.

We determined total organic carbon (%TOC of dry matter) and bulk stable isotope composition for acid insoluble organic matter (AIOM) and EPS extracted from SLM sediments with a Carlo-Erba NAN2500 Series-II Elemental Analyzer coupled to a continuous flow Thermo-Finnigan Delta+ XL isotope ratio mass spectrometer (IRMS). NIST 8573 and NIST 8574 were used as calibration standards and a low carbon, bulk Antarctic sediment sample (JGC20C) as a working standard. Analytical uncertainty, expressed as ±1 standard deviation of replicate measurements of the working standard, was ± 0.02% for percent TOC and 0.5‰ for δ¹³C during the period over which we analyzed AIOM samples and ± 0.86% for percent TOC and 0.1‰ for δ¹³C during the period over which we analyzed EPS samples.

Samples were prepared for scanning transmission X-ray spectroscopy (STXM) at Diamond Light Source (UK) I08 beamline in the organic geochemistry lab at Florida State University. A dried EPS pellet was redissolved in a small amount of ultra-pure water and a ~10 µL aliquot was pipetted onto a Si_3_N_4_ TEM grid and allowed to air dry under a laminar flow hood. The sample was placed into a custom TEM grid holder at Diamond Light Source and mounted on the beamline at I08. For the EPS sample, we imaged and spectrally mapped larger areas (200 µm^2^) at a resolution of 2000 nm pixel^-1^, to identify regions (~30 µm^2^) of high interest for subsequent collection of C µ-NEXAFS energy stacks. C intensity difference maps were generated by subtracting energy data at 280 eV from 290 eV (pre and post C K-edge). Three regions of interest identified from these difference maps were scanned over the C K-edge from 280 eV to 320 eV, at a spectral resolution of 0.1 – 1 eV (with higher resolution closer to the C K-edge between 282 and 292 eV), and dwell time of 10 msec and at a spatial resolution of 500 nm. Pixels were binned to generate representative C K-edge Near-Edge X-ray Absorption Fine Structure (NEXAFS) for regions of interest using PCA/cluster analysis in Mantis [21]. Carbon functional group peaks on the C K-edge were deconvoluted using MagicPlot Pro using expected functional group peak positions from the literature [22-24].

EPS was dissolved in 10 ml Milli-Q water and mixed. The sample was transferred to a quartz glass cuvette with a 10 mm pathlength and analyzed for absorbance and fluorescence using an Aqualog spectrofluorometer (Horiba Scientific) that uses a 150 W xenon arc lamp excitation source. The dissolved EPS samples were scanned in total fluorescence mode to obtain excitation-emission matrices (EEMs), exciting from 240-500 nm at 3 nm increments and monitoring emission from 250-800 nm at 5 nm increments. An EEM taken of a NIST certified water blank measured under identical conditions was subtracted from each individual EEM. EEMs were obtained using a maximum 20 s integration time to optimize the instrument signal to noise ratio.

*Sedimentological and geochemical analysis*

To measure sediment water content, cutoff 3 mL syringes were used to sample 1 or 2 mL of sediment, and the entire sediment volume was transferred into a pre-weighed medical cup with lid. The wet sediment weight of known volume was recorded, then the sediment was dried at 95°C until the masses stopped decreasing (~2 days) and dry weight recorded. All measurements in Supplemental Data File 1 represent dry weight values.

Highly reactive nanoparticle iron was extracted from sediments using a well characterized two-step chemical leach [25, 26]. An ascorbate solution was used to extract amorphous/poorly crystalline freshly precipitated iron (oxyhyr)oxides (previously calibrated to freshly precipitated 2-line ferrihydrite) and particle surface bound Fe^2+^ [27, 28]. A subsequent dithionite leach on the same sediment was used to remove highly reactive but more crystalline iron nanoparticles, such as aged 2-line ferrihydrite, 6-line ferrihydrite, nano-goethite and nano-hematite [26, 29]. The ascorbate solution was a deoxygenated solution of 0.17 M sodium citrate and 0.6 M sodium bicarbonate to which ascorbic acid was added (to a final concentration of 0.057 M). The pH of the ascorbate solution was subsequently adjusted to 7.5 using additional ascorbic acid. The dithionite solution was a solution of 0.3 M sodium dithionite, adjusted to pH 4.8 with a buffer solution of 0.35 M acetic acid and 0.2 M sodium citrate. For the extractions, 30 mg of sediment was accurately weighed into a 10 mL PP centrifuge tube, 10 mL of ascorbate solution was added, and the mixture was shaken for 24 hours in the dark at room temperature. The solution was then centrifuged at 5000 rpm for 10 minutes and the supernatant removed and filtered through a 0.2 um PES syringe filter into a clean 50 mL centrifuge tube. The remaining sediment was rinsed with 10 mL of ultra-pure water (18.2 MΩ cm^-1^), centrifuged for 10 minutes, and filtered into the centrifuge tube with initial ascorbate extractant. Ten mL of dithinoite solution was then added to the sediment, shaken for 2 h in the dark at room temperature, and filtered into a clean 50 mL centrifuge tube through a 0.2 um PES syringe filter. Sediment extracts were diluted to 50 mL and acidified with ultra-trace metal grade HNO_3_ (lab distilled) to 0.2 % v/v. Before analysis, all sediment extracts were diluted 1 in 20 with a 2 % HNO_3_ solution. Samples were analyzed on an Agilent 7500cs quadrupole ICP-MS with He in the collision cell to reduce polyatomic interferences from ArO on ^56^Fe. An internal standard solution of 10 ppb Sc and Y was mixed with samples in-line prior to introduction into the ICP-MS to correct for drift and matrix effects. Accuracy and precision were determined from replicate measurements of a known standard and were always better than ±5%.

Solid phase sulfides were distilled from the sediment with a standard two-step sulfide extraction [30]. Two operational definitions of solid phase sulfide were defined given the solutions used to separate sulfide from the sediment. First, the acid volatile sulfide (AVS) is the sulfide released during cold distillation with 6M HCl and is considered to represent sulfide from the FeS pool. Second, the chromium reducible sulfide (CRS) is the sulfide released during boiling distillation with 2M CrCl_2_ and 2M HCl and is considered to represent sulfide from the FeS_2_ pool. Sediment was weighed into round-bottom reaction flasks with an inlet for N_2_ and a valve port for adding reagents. The flasks were connected to distillation columns jacketed with recirculating cold water to keep hot, acidic, water vapor from being transported to the trapping solution. Volatile sulfide travelled through a 0.1 M citrate buffer (pH 4) and then into a 5% zinc acetate trap, where a ZnS precipitate formed, trapping the sulfide. The first step of the fractional distillation was a 1.5 h incubation with 10 mL of 6M HCl to extract and trap the S from FeS at room temperature. After the FeS distillation step, a new 5% Zinc acetate trap was installed and the 2M CrCl_2_ solution added and heated until the solution was lightly boiling. The second distillation step ran for 1.5 h and constant light boiling. Sulfide trapped as ZnS during distillation was quantified spectrophotometrically using the diamine reaction [31]. Briefly, ZnS was suspended by vortexing and diluted 1:2 with water for the AVS samples and 1:100 for the CRS samples. Diamine (80 µL) was added to each of the samples and standards, shaken, and stored in the dark for 30 min to allow for color development. After color development, 300 µL of samples and standards were transferred to a 96-well plate and absorbance measured on a plate reader at 672 nm. A sulfide standard curve was used to calculate the concentration of ZnS in each trap, then multiplied by the volume of the trap solution for the total mass of sulfide extracted from the sediment. Sediment sulfide concentration was calculated by dividing the mass of sulfide extracted by the mass of sediment.

Cutoff 3 mL syringes were inserted into the sediment to remove a 3 mL plug of sediment and then capped with a butyl rubber stopper. The sediment-filled syringes were then transferred to the chemistry laboratory where 5 µL of 100 kBq of carrier-free ^35^SO_4_^2-^ using a glass syringe. The syringes were incubated with the tracer for 12 hours. After the incubation period, the syringes with sediment were frozen at -20ºC to stop sulfate reduction. The sediment was shipped frozen to Aarhus University where it was processed. The sediment in the syringes was extruded into round bottom flasks with 10 mL of 10% zinc acetate immediately after removal from the freezer and allowed to thaw in the zinc acetate solution. The ^35^SO_4_^2-^ reduced to ^35^S-sulfide was recovered from the sediment using the cold chromium distillation method [32, 33]. This method recovers the total reduced inorganic sulfur pool as ^35^S-sulfide in a 5% zinc acetate trap. The radioactivity present in the 5% zinc acetate trap was quantified using scintillation counting (20 min each sample or at least 2000 counts) and counts were converted to a sulfate reduction rate accordingly [30].

*Nucleic Acid Extraction, Amplification, and Sequencing*

All experimental manipulations for the molecular analysis were conducted in a class 100 laminar flow hood. Genomic DNA was extracted from thawed sediments using the same method (Qiagen PowerSoil DNA Isolation kit) used to analyze samples from SLW [10, 11] and the WGZ [34]. From 0.5 to 1 g (wet weight) of multicore or bulk sediment was added to the bead beating tubes. For samples recovered from the gravity cores, methods were optimized to extract from 2.5 to 3.5 g of sediment (wet weight). Tubes were placed in a Mixer Mill 400 (Retsch, Inc.) and homogenized at 20 Hz for 10 minutes. DNA was extracted according to the manufacturer’s protocol for the bulk and multicore samples. Modifications for the gravity core samples included using 5 mL centrifuge tubes, the beads from two PowerSoil bead tubes, and solution volumes for C1, C2, C3, and C4 were doubled. After mixing solution C4 with the DNA containing supernatant, the total volume obtained was applied to a single spin column in the PowerSoil extraction kit. All samples were eluted in 75 to 100 µL of warmed elution buffer. Procedural controls that consisted of blank filters and the same materials and reagents as the sediment samples were included in each extraction to monitor potential sources of extraneous cell and DNA contamination.

PCR was used to amplify the V4 region of the 16S rRNA gene in a 50 µL volume that contained the following components: 2.5 U of AmpliTaq Gold DNA Polymerase (Invitrogen), 1x PCR Gold Buffer, 3.5 mM MgCl_2_, 200 µM deoxynucleotides (dNTPS), 200 µM of each primer [35], and 12 to 98 pg of DNA template. The amplification conditions initiated with 35 to 42 cycles (95 ºC for 15 s, 50 ºC for 45 s, and 72 ºC for 1 min). The final step was a terminal elongation step at 72 ºC for 10 min. The PCR products generated were evaluated by electrophoresis through a 1% agarose gel and quantified using the high sensitivity dsDNA kit (Qubit). Negative controls for the PCR and DNA extraction were included in each experiment and consisted of the same materials and reagents used to amplify DNA from the samples.

Amplicons of the correct size were cleaned using the Qiaquick PCR Clean-Up Kit (Qiagen), pooled in equal concentrations of 5.0 ng µL^-1^, and sequenced at the University of Florida NextGen DNA Sequencing Core. For each sequencing run, amplicons generated using the ZymoBIOMICS^TM^ Microbial Community Standard (Zymo Research) as template were also sequenced in parallel with the samples and procedural controls. Sequencing was performed on an Illumnia MiSeq platform using the 2 x 300 v3 run format. The sequencing data are available in the Sequence Read Archive of NCBI under the projects PRJNA790995 (SLM data), PRJNA244335 (SLW data), and PRJNA869494 (WGZ data).

*ASV Determination and Statistical Analysis*

Adapter sequences obtained from the pooled libraries were trimmed from the paired-end reads using Scythe, a Bayesian adapter trimmer available at Vince Buffalo’s GitHub repository. In R version 4.1.1, DADA2 v1.16.0 was used to process data from the V4 region of the 16S rRNA gene. Individual sequencing reads less than 250 bp were removed using the command “filterAndTrim” with a “maxN” score of 0, “truncQ” score of 2, and “truncLen” score of 250 bp forward and 250 bp reverse [36]. Taxonomy was assigned using the SILVA database v138 training set and based on the Naïve Bayesian Classifier algorithm in DADA2 [37]. Contaminants identified in the procedural controls were removed using the ‘decontam’ package within R [38] and sequences identified as eukaryotic were also removed.

Diversity and richness estimates (Shannon, Chao1, Inverse Simpson, and Simpson) were calculated using phyloseq (v1.36.0; [39]) in R. Spearman’s rank order correlation tests were used for pairwise comparisons across physical, biogeochemical, and microbiological variables within the sediment profile. Non-metric multidimensional scaling (NMDS) plots based on Bray-Curtis dissimilarity matrices were calculated in R using the vegan (v2.5.7; [40]) package. Multivariate analysis was applied using redundancy analysis (rda fuction) in the vegan package. Statistical significance among samples in the RDA and NMDS analysis was evaluated using the adonis function. In the RDA, environmental variables and species were co-plotted based on Euclidean distance. Statistical significance (α = 0.05) of environmental variables to community composition were determined using a Mantel test based on Spearman’s correlation method. ASVs used for the analyses in Figures 5 & S5 were aligned based on 16S rRNA secondary structure using SILVA Incremental Aligner (SINA) [41]. The alignments were analyzed in MEGA11 to generate maximum likelihood phylogenies using the Bootstrap (100 replications) and Tamura-Nei methods [42-44].

**Supplementary Results:**

*Physical Properties of the Sediment*

Based on appearance, physical properties, and density changes observed under computed tomography imaging, continuous lithological features observed in the shallow multicores and deeper freefall cores were aligned to produce a composite depth scale. Unless otherwise specified, all sediment depths refer to the composite depth scale described by Venturelli et al. [13]. The sediment water content in unit I ranged from 0.603 to 0.819, dropping abruptly to values as low as 0.384 at the transition between units I and II (11 to 15 cm) and continuing to decrease with depth to 37 cm (Fig. 1b). At depths below 37 cm, the water content was relatively constant at ~0.2. The specific conductance profile (Fig. 1b) indicates fresher pore waters in the surficial sediments (360 µS cm^-1^) that becomes more brackish with depth, increasing linearly in the samples to 5390 µS cm^-1^ at 200 cm.

*Sedimentary sulfide*

Chromium reducible sulfide (CRS), which comprises both S^0^ and FeS_2_, was lowest at depths between 5 and 11 cm (<92 µg S g^-1^), but within unit I, its concentration was highest at the surface (Fig. 1e). At the transition between units I and II, CRS increases rapidly by ~twofold (71 to 133 µg S g^-1^) and then decreases to 91 µg S g^-1^ at a depth of 21 cm. Between depths of 21 to 200 cm, the CRS concentration increased linearly, and a maximum concentration of 211 µg S g^-1^ is observed in unit III at 153 cm (Fig. 1e). Acid volatile sulfide (AVS) is positively and significantly correlated with oxygen (r_s_ = 0.59, n = 24, p < 0.005). The lack of sulfate reducing activity implies hydrogen sulfide may not have been present and that the AVS was probably predominantly FeS. The profile for AVS concentration followed a similar trend to CRS (Fig. 1f): the AVS concentration was lowest at oxic depths between 15 and 30 cm (<0.015 µg S g^-1^), these values doubled to 0.030 µg S g^-1^ at the anoxic transition, and the highest AVS concentrations of ~0.06 and ~0.10 µg S g^-1^ were measured in samples from anoxic depths of units III and IV, respectively (Fig. 1e).

*Total organic carbon*

The data for total organic carbon (TOC) concentration and bulk stable isotopic composition of organic matter from Venturelli et al. [13] are plotted in Figure 1f and 1i, respectively. δ^13^C values for TOC that range from -31.1 to -18.4‰ [13] are consistent with the bulk of the sedimentary organic matter being derived from marine DIC. The range of values from stable carbon isotope composition are typical of open ocean organic matter compared to lighter values that are similar to modern day Ross Sea organic matter [45]. The higher TOC concentrations observed in unit I (0.39 ± 0.08%; mean± standard deviation) are statistically higher (Mann-Whitney *U* = 13.5; n_1_ = 13, n_2_ = 45; p < 0.001) than those observed in the underlying units. TOC is significantly positively correlated to the concentration of oxygen (*r_s_* = 0.84, n = 37, p < 0.001), and at depths >13 cm, the concentration is relatively invariable at 0.14 ± 0.01% (Fig. 1f).

*Characterization of EPS*

The estimated carbon content of EPS extracted from the sediment samples ranges from 0.001 to 22 μg C g^-1^ and positively and significantly correlates to sediment TOC concentration (*r_s_* = 0.69, n = 17, p > 0.005). As per total EPS carbon, the carbohydrate and DNA components of the EPS showed their highest concentrations in the surficial depths of unit I and decreased with depth (Fig. 1h). C K-edge NEXAFS were homogenous in all regions of interest analyzed. The NEXAFS displayed characteristic peaks at ~285 eV, 287.8 eV and 288.5 eV (Fig. S1a), which are indicative of aromatic, aliphatic and carboxylic functional groups. Peak deconvolution of spectra indicates that the largest contribution comes from aromatic moieties, followed by aliphatic moieties and then carboxylic-ester-acetal moieties, with a minor contribution from ketones, phenols, and amine moieties (Fig. S1b). EPS NEXAFS also displayed distinctive K L-edge peaks at ~295 and 297.5 eV.

To examine the source of carbon of the extracted EPS, we measured δ^13^C of EDTA-extracted sediments (assumed to be EPS), procedural blanks, and EDTA (-25.14‰ relative to VPDB). This enabled us to determine that δ^13^C values for EDTA-extracted sediments primarily reflect that of EPS and not the EDTA used during the extraction. Support for no obvious contamination of EPS by EDTA extractant was provided by analysis of an EDTA standard using C K-edge NEXAFS, which highlighted the EDTA functional groups ~286.4 eV (close to the expected location of the amine functional group) and ~288.2 eV (indicative of the carboxyl functional group; Fig. S1c). The EDTA NEXAFS spectra is distinctive from the EPS NEXAFS (Fig. S2), particular the lack of N-bearing moieties in the latter.

The δ^13^C values of the EPS in the sediment are fairly constant with depth and range from -26.6‰ (35 cm) to -25.7‰ (86 cm; Fig. 1i), with percent carbon content ranging from 2.7 to 14.2%. The overlying water column (samples that passed through 0.2 μm pore size filter) had the lowest δ^13^C value of -30.0‰ and the highest carbon content (70%). Visual inspection of the extracted EPS pellets revealed additional fine clays in the sediment samples compared to the water column, which would influence the carbon weight percentage estimate. EPS δ^13^C values are generally lower than bulk TOC δ^13^C at most depths, the exceptions being at depths of 31 and 140 cm that had EPS nearly indistinguishable from bulk TOC measurements. On average, the difference between δ^13^C values for TOC and EPS are larger at oxic sediment depths <30 cm. The δ^13^C values for EPS are within the range for bulk organic matter in SLM (-26.9 to -21.0‰; Fig. 1i). Thermal and chemical decomposition of the SLM organic matter mixture shows that the radiocarbon-bearing (i.e., most recently deposited) part of this mixture is characterized by a significantly lighter δ^13^C value than bulk TOC -33.9 to -30.41‰ [13].

*Sequence analysis*

Sequencing of 16S rRNA gene amplicons from 58 multiplexed SLM sediment samples generated a total of 16,181,103 raw reads. After quality filtering, exclusion of chimeras and contaminants, and the removal of sequences that classified as 18S rRNA genes, the remaining 11,710,854 sequences were used to identify a total of 5,605 ASVs in the samples. In comparison, 1,539 and 908 ASVs were identified in the SLW (n=4) and WGZ (n=5) sediment samples, respectively. The ‘decontam’ R package identified 41 ASVs as contaminants within 22 of the 58 samples. In these samples, the contaminant sequences represented a maximum of 0.18% (8 to 20,911 sequences) of the total sequences in the samples. The largest number of reads (average of 322,076 per sample) and ASVs (3,218) were observed in unit I of SLM (Table 1). Samples from units II, III, and IV have 35% to 49% fewer reads and 14% to 63% fewer ASVs when compared to unit I.

For each sequencing run, a mock community was co-analyzed and 99.9% of the reads obtained were affiliated with species in the standard. Linear regression analysis of the relative abundance of taxa observed in the mock community showed that there was good reproducibility between the independent sequencing runs (r^2^ = 0.95; Fig S3A). Observed versus theoretical taxon abundances in the mock community were similar (r^2^ = 0.40 and 0.35) and the largest residuals are associated with two of the *Firmicutes* species (Fig S3B). Specifically, the abundance of *Lactobacillus* *fermentum* was underestimated in both sequencing runs by ~two-fold whereas *Bacillus subtilis* was overestimated by ~50% in one of the sequencing runs.

**SALSA Science Team (full list):**

Carlo Barbante^1^, Joel D. Barker^2^, Mark Bowling^3^, Justin Burnett^4^, Timothy Campbell^5^, Brent C. Christner^6^, Billy Collins^7^, Christina L. Davis^6^ , Cindy Dean^8^, John E. Dore^8^, Dennis Duling^3^, Helen A. Fricker^9^, Alan Gagnon^10^, Christopher Gardner^11^, Dar Gibson^3^, Chloe Gustafson^12^, David Harwood^3,13^, Jon R. Hawkings^14^ , Jonas Kalin^3^, Kathy Kasic^15^, Ok-Sun Kim^16^, Edwin Krula^3^, Amy Leventer^17^, Wei Li^8^, W. Berry Lyons^11^, Patrick McGill^15^, James McManis^3^, David McPike^3^, Alexander B. Michaud^18^, Anatoly Mironov^3^, Molly Patterson^19^, John C. Priscu^20^, Graham Roberts^3^, Brad E. Rosenheim^21^, James Roth^3^, Matthew R. Siegfried^22^, Mark L. Skidmore^5^, August Steigmeyer^5^, Cathy Trainor^7^, Martyn Tranter^23^, Trista J. Vick-Majors^24^, Ryan A. Venturelli^25^, John Winans^3^, and Bob Zook^3^.

^1^Institute for the Dynamics of Environmental Processes, University Ca’Foscari, Venice, Italy

^2^School of Earth and Environmental Sciences, University of Minnesota, Minneapolis, MN, USA

^3^Antarctic Science Management Office, University of Nebraska – Lincoln, NE, USA

^4^Applied Physics Lab, University of Washington, Seattle, WA, USA

^5^Department of Earth Sciences, Montana State University, Bozeman, MT, USA

^6^Department of Microbiology and Cell Science, University of Florida, Gainesville, FL, USA

^7^School of Film and Photography, Montana State University, Bozeman, MT, USA

^8^Department of Land Resources and Environmental Sciences, Montana State University, Bozeman, MT, USA

^9^Scripps Institution of Oceanography, University of California San Diego, CA, USA

^10^Woods Hole Oceanographic Institution, Falmouth, MA, USA

^11^School of Earth Sciences, Byrd Polar and Climate Research Center, The Ohio State University, Columbus, OH, USA

^12^Department of Earth and Environmental Sciences, Columbia University, New York, NY, USA

^13^Department of Earth and Atmospheric Sciences, University of Nebraska – Lincoln, NE, USA

^14^Department of Earth and Environmental Science, University of Pennsylvania, Philadelphia, PA, USA

^15^Film Program, Communication Studies, California State University, Sacramento, CA, USA

^16^Division of Polar Life Sciences, Korea Polar Research Institute, Incheon, South Korea

^17^Department of Geology, Colgate University, Hamilton, NY, USA

^18^Center for Geomicrobiology, Aarhus University, Aarhus, DK and present address: Bigelow Laboratory for Ocean Sciences, East Boothbay, ME, USA

^19^Department of Geological Sciences and Environmental Studies, Binghamton University, Vestal, NY, USA

^20^Polar Oceans Research Group, Sheridan, MT, USA

^21^College of Marine Sciences, University of South Florida, St. Petersburg, FL, USA

^22^Department of Geophysics, Colorado School of Mines, Golden, CO, USA

^23^Department of Environmental Science, Aarhus University, Aarhus, Denmark

^24^Department of Biological Sciences, Michigan Technological University, Houghton, MI, USA

^25^Department of Geology and Geological Engineering, Colorado School of Mines, Golden, CO, USA

**Reference:**

1. Siegfried M, Fricker H, Carter S, Tulaczyk S. Episodic ice velocity fluctuations triggered by a subglacial flood in West Antarctica. Geophysical Research Letters. 2016;43(6):2640-8.

2. Siegfried M, Fricker H. Illuminating Active Subglacial Lake Processes With ICESat-2 Laser Altimetry. Geophysical Research Letters. 2021;48(14).

3. Siegfried MR, Venturelli RA, Patterson MO, Arnuk W, Campbell TD, Gustafson CD, et al. The life and death of a subglacial lake in West Antarctica. (submitted).

4. Blythe D, Duling D, Gibson D. Developing a hot-water drill system for the WISSARD project: 2. In situ water production. Annals of Glaciology. 2014;55(68):298-310.

5. Burnett J, Rack FR, Blythe D, Swanson P, Duling D, Gibson D, et al. Developing a hot-water drill system for the WISSARD project: 3. Instrumentation and control systems. Annals of Glaciology. 2014;55(68):303-10.

6. Rack F, Duling D, Blythe D, Burnett J, Gibson D, Roberts G, et al. Developing a hot-water drill system for the WISSARD project: 1. Basic drill system components and design. Annals of Glaciology. 2014;55(68):285-97.

7. Priscu J, Achberger A, Cahoon J, Christner B, Edwards R, Jones W, et al. A microbiologically clean strategy for access to the Whillans Ice Stream subglacial environment. Antarctitc Science. 2013;25(5):637-47.

8. Michaud A, Vick-Majors T, Achberger A, Skidmore M, Christner B, Tranter M, et al. Environmentally clean access to Antarctic subglacial aquatic environments. Antarctic Science. 2020:1-12.

9. Priscu JC, Kalin J, Winans J, Campbell T, Siegfried MR, Skidmore M, et al. Scientific access into Mercer Subglacial Lake: scientific objectives, drilling operations and initial observations. Annals of Glaciology. 2021;62(85-86):340-52.

10. Christner BC, Priscu JC, Achberger AM, Barbante C, Carter SP, Christianson K, et al. A microbial ecosystem beneath the West Antarctic ice sheet. Nature. 2014;512(7514):310-3.

11. Achberger A, Christner B, Michaud A, Priscu J, Skidmore M, Vick-Majors T, et al. Microbial Community Structure of Subglacial Lake Whillans, West Antarctica. Frontiers in Microbiology. 2016;7.

12. Michaud A, Dore J, Achberger A, Christner B, Mitchell A, Skidmore M, et al. Microbial oxidation as a methane sink beneath the West Antarctic Ice Sheet. Nature Geoscience. 2017;10(8):582-+.

13. Venturelli RA, Boehman B, Davis C, Hawkings JR, Johnston SE, Gustafson CD, et al. Constraints on the timing and extent of deglacial grounding line retreat in West Antarctica from subglacial sediments. (submitted).

14. Kallmeyer J, Smith DC, Spivack AJ, D'Hondt S. New cell extraction procedure applied to deep subsurface sediments. Limnology and Oceanography: Methods. 2008;6(6):236-45.

15. Pan D, Morono Y, Inagaki F, Takai K. An Improved Method for Extracting Viruses From Sediment: Detection of Far More Viruses in the Subseafloor Than Previously Reported. Front Microbiol. 2019;10:878.

16. Moore RA, Bomar C, Kobziar LN, Christner BC. Wildland fire as an atmospheric source of viable microbial aerosols and biological ice nucleating particles. ISME J. 2021;15(2):461-72.

17. Battin T, Wille A, Sattler B, Psenner R. Phylogenetic and functional heterogeneity of sediment biofilms along environmental gradients in a glacial stream. Applied and Environmental Microbiology. 2001;67(2):799-807.

18. Klock J-H, Wieland A, Seifert R, Michaelis W. Extracellular polymeric substances (EPS) from cyanobacterial mats: characterisation and isolation method optimisation. Marine Biology. 2007;152(5):1077-85.

19. Miyatake T, Moerdijk-Poortvliet T, Stal L, Boschker H. Tracing carbon flow from microphytobenthos to major bacterial groups in an intertidal marine sediment by using an in situ C-13 pulse-chase method. Limnology and Oceanography. 2014;59(4):1275-87.

20. Albalasmeh A, Berhe A, Ghezzehei T. A new method for rapid determination of carbohydrate and total carbon concentrations using UV spectrophotometry. Carbohydrate Polymers. 2013;97(2):253-61.

21. Lerotic M, Mak R, Wirick S, Meirer F, Jacobsen C. MANTiS: a program for the analysis of X-ray spectromicroscopy data. Journal of Synchrotron Radiation. 2014;21:1206-12.

22. Solomon D, Lehmann J, Kinyangi J, Liang B, Heymann K, Dathe L, et al. Carbon (1s) NEXAFS Spectroscopy of Biogeochemically Relevant Reference Organic Compounds. Soil Science Society of America Journal. 2009;73(6):1817-30.

23. Le Guillou C, Bernard S, De la Pena F, Le Brech Y. XANES-Based Quantification of Carbon Functional Group Concentrations. Analytical Chemistry. 2018;90(14):8379-86.

24. Bonneville S, Delpomdor F, Preat A, Chevalier C, Araki T, Kazemian M, et al. Molecular identification of fungi microfossils in a Neoproterozoic shale rock. Science Advances. 2020;6(4).

25. Raiswell R, Benning L, Tranter M, Tulaczyk S. Bioavailable iron in the Southern Ocean: the significance of the iceberg conveyor belt. Geochemical Transactions. 2008;9.

26. Raiswell R, Vu H, Brinza L, Benning L. The determination of labile Fe in ferrihydrite by ascorbic acid extraction: Methodology, dissolution kinetics and loss of solubility with age and de-watering. Chemical Geology. 2010;278(1-2):70-9.

27. Raiswell R, Hawkings J, Eisenousy A, Death R, Tranter M, Wadham J. Iron in Glacial Systems: Speciation, Reactivity, Freezing Behavior, and Alteration During Transport. Frontiers in Earth Science. 2018;6.

28. Hyacinthe C, Bonneville S, Van Cappellen P. Reactive iron(III) in sediments: Chemical versus microbial extractions. Geochimica Et Cosmochimica Acta. 2006;70(16):4166-80.

29. Raiswell R, Canfield D, Berner R. A comparison of iron extraction methods for the determination of degree of pyritisation and the recognition of iron-limited pyrite formation. Chemical Geology. 1994;111(1-4):101-10.

30. Fossing H, Jorgensen B. Measurement of bacterial sulfate reduction in sediments - evaluation of a single-step chromium reduction method. Biogeochemistry. 1989;8(3):205-22.

31. Cline J. Spectrophotometric determination of hydrogen sulfide in natural waters. Limnology and Oceanography. 1969;14(3):454-&.

32. Kallmeyer J, Ferdelman T, Weber A, Fossing H, Jorgensen B. A cold chromium distillation procedure for radiolabeled sulfide applied to sulfate reduction measurements. Limnology and Oceanography-Methods. 2004;2:171-80.

33. Roy H, Weber H, Tarpgaard I, Ferdelman T, Jorgensen B. Determination of dissimilatory sulfate reduction rates in marine sediment via radioactive S-35 tracer. Limnology and Oceanography-Methods. 2014;12:196-211.

34. Achberger A. Structure and functional potential of microbial communities in Subglacial Lake Whillans and at the Ross Ice Shelf Grounding Zone, West Antarctica: Louisiana State University; 2016.

35. Caporaso J, Lauber C, Walters W, Berg-Lyons D, Huntley J, Fierer N, et al. Ultra-high-throughput microbial community analysis on the Illumina HiSeq and MiSeq platforms. Isme Journal. 2012;6(8):1621-4.

36. Callahan BJ, McMurdie PJ, Rosen MJ, Han AW, Johnson AJ, Holmes SP. DADA2: High-resolution sample inference from Illumina amplicon data. Nat Methods. 2016;13(7):581-3.

37. Wang Q, Garrity GM, Tiedje JM, Cole JR. Naive Bayesian classifier for rapid assignment of rRNA sequences into the new bacterial taxonomy. Appl Environ Microbiol. 2007;73(16):5261-7.

38. Davis NM, Proctor DM, Holmes SP, Relman DA, Callahan BJ. Simple statistical identification and removal of contaminant sequences in marker-gene and metagenomics data. Microbiome. 2018;6(1):226.

39. McMurdie PJ, Holmes S. Shiny-phyloseq: Web application for interactive microbiome analysis with provenance tracking. Bioinformatics. 2015;31(2):282-3.

40. Oksanen J, Blanchet FG, Friendly M, Kindt R, Legendre P, McGlinn D, Minchin PR, O'Hara RB, Simpson GL, Solymos P, Stevens MHH, Szoecs E, and Wagner H (2020). vegan: Community Ecology Package. R package version 2.5-7. https://CRAN.R-project.org/package=vegan.

41. Pruesse E, Peplies J, Glockner F. SINA: Accurate high-throughput multiple sequence alignment of ribosomal RNA genes. Bioinformatics. 2012;28(14):1823-9.

42. Kumar S, Stecher G, Li M, Knyaz C, Tamura K. MEGA X: Molecular Evolutionary Genetics Analysis across Computing Platforms. Molecular Biology and Evolution. 2018;35(6):1547-9.

43. Stecher G, Tamura K, Kumar S. Molecular Evolutionary Genetics Analysis (MEGA) for macOS. Mol Biol Evol. 2020.

44. Tamura K, Nei M. Estimation of the number of nucleotide substitutions in the control region of mitochondrial-DNA in humans and chimpanzees. Molecular Biology and Evolution. 1993;10(3):512-26.

45. Villinski J, Hayes J, Brassell S, Riggert V, Dunbar R. Sedimentary sterols as biogeochemical indicators in the Southern Ocean. Organic Geochemistry. 2008;39(5):567-88.

46. Latham K, Simone M, Dose W, Allen J, Donne S. Synchrotron based NEXAFS study on nitrogen doped hydrothermal carbon: Insights into surface functionalities and formation mechanisms. Carbon. 2017;114:566-78.

**Supplemental Tables**

**Table S1: Sediment cores and samples from SLM analyzed in this study**

| Core designation | Short Name | Core/Sample Type | Analyses performed |
| --- | --- | --- | --- |
| SLM-1801-01UW-A | MC1A | multicore | TOC concentration |
| SLM-1801-01UW-B | MC1B | multicore | water content, conductivity, methane concentration, TOC concentration, solid phase sulfide, ion concentration, cell enumeration, 16S rRNA gene sequencing, EPS quantification and characterization |
| SLM-1801-01UW-C | MC1C | multicore | TOC concentration, reactive iron quantification, cell enumeration, 16S rRNA gene sequencing |
| SLM-1801-02UW-A | MC2A | multicore | oxygen concentration |
| SLM-1801-02UW-B | MC2B | multicore | Pore water ion concentration |
| SLM-1801-02UW-C | MC2C | multicore | oxygen concentration |
| SLM-1801-04UW-C | MC4C | multicore | cell enumeration, 16S rRNA gene sequencing |
| SLM-1801-01FF | 01FF | gravity core | conductivity, TOC concentration, methane concentration, reactive iron quantification, water content, solid phase sulfide, cell enumeration, 16S rRNA gene sequencing |
| SLM-1801-02FF | 02FF | gravity core | conductivity, TOC concentration, reactive iron, quantification, water content, cell enumeration, 16S rRNA gene sequencing, EPS quantification and characterization |
| SLM-WTS-LV-BS3 | BS3 | bulk sediment | 16S rRNA gene sequencing |
| SLM-WTS-LV-BS4 | BS4 | bulk sediment | 16S rRNA gene sequencing |

**Table S2: Description of ASVs shared among sediments from SLM, SLW, and WGZ**

| **ASV** | **Description** | **Accession Number** | **Percent ID** |
| --- | --- | --- | --- |
| ASV_2 | *Thiobacillus thioparus* | NR_117864.1 | 99% |
| ASV_18 | *Sulfuricaulis limicola* | NR_147747.1 | 92% |
| ASV_26 | *Methylotenera oryzisoli* | NR_175447.1 | 100% |
| ASV_34 | *Herbaspirillum robiniae* | NR_163661.1 | 100% |
| ASV_42 | Unclassified Thermodesulfovibrionia |  | <90% |
| ASV_44 | *Rhodoferax ferrireducens* | NR_074760.1 | 100% |
| ASV_52 | Unclassified Actinobacteriota |  | <90% |
| ASV_75 | *Sphingopyxis solisilvae* | NR_157002.1 | 96% |
| ASV_87 | *Porticoccus hydrocarbonoclasticus* | NR_118247.1 | 94% |
| ASV_92 | Unclassified Thermodesulfovibrionia |  | <90% |
| ASV_94 | *Ignavibacterium album* | NR_074698.1 | 96% |
| ASV_112 | *Lutibacter maritimus* | NR_116738.1 | 98% |
| ASV_137 | *Cloacibacterium rupense* | NR_114274.1 | 100% |
| ASV_153 | *Ohtaekwangia koreensis* | NR_117435.1 | 94% |
| ASV_187 | *Nitrosospira multiformis* | NR_074736.1 | 95% |
| ASV_190 | *Sideroxydans lithotrophicus* | NR_074731.1 | 97% |
| ASV_191 | Unclassified Bacteroidota |  | <90% |
| ASV_211 | *Lutibacter oceani* | NR_146841.1 | 95% |
| ASV_221 | *Maribellus maritimus* | NR_181847.1 | 94% |
| ASV_226 | Unclassified Caldatribacteriota |  | <90% |
| ASV_255 | *Leptolinea tardivitalis* | NR_040971.1 | 93% |
| ASV_278 | Unclassified Chloroflexi |  | <90% |
| ASV_329 | Unclassified Thermodesulfovibrionia |  | <90% |
| ASV_402 | Unclassified Actinobacteriota |  | <90% |
| ASV_440 | Unclassified Bacteria |  | <90% |
| ASV_623 | Unclassified Chloroflexi |  | <90% |
| ASV_670 | Unclassified Chloroflexi |  | <90% |
| ASV_806 | Unclassified Actinobacteriota |  | <90% |
| ASV_1136 | Unclassified Nitrospinota |  | <90% |
| ASV_1241 | Unclassified Anaerolineaceae |  | <90% |
| ASV_1737 | *Thermomarinilinea lacunifontana* | NR_132293.1 | 92% |
| ASV_2568 | Unclassified Chloroflexi |  | <90% |

**Supplemental Figures**


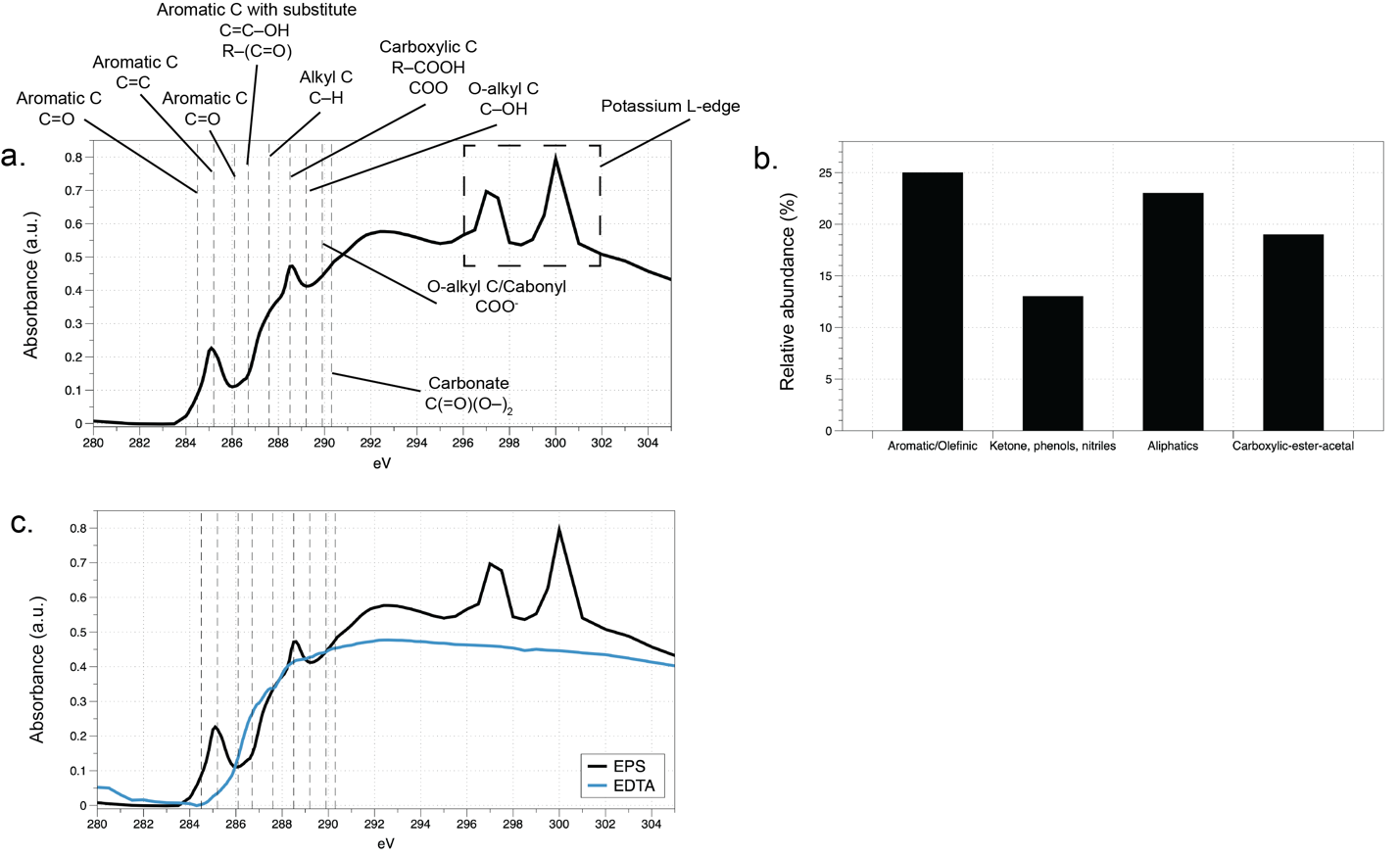


**Figure S1:** (a) NEXAFS spectra of extracted EPS material on the C K-edge with key functional group transitions indicated. Note that the K L-edge peaks indicate some inorganic material was also extracted from the sediments. (b) relative abundance of functional groups in EPS after peak deconvolution and spectra fitting as per Le Guillou et al. [23]. (c) comparison of EPS NEXAFS spectra (in black) and background EDTA NEXAFS spectra (the extractant; in blue), with characteristic functional group locations as per (a). Note predominant peak locations for EDTA functional groups are at ~287.3 eV (where the C-N amine functional group transition, 1s→π* (C–NHx), is likely to occur; [46]) and ~288.2 eV (carboxylic functional group; 1s→π* (C=O_carboxylic acid/carboxylate_)).


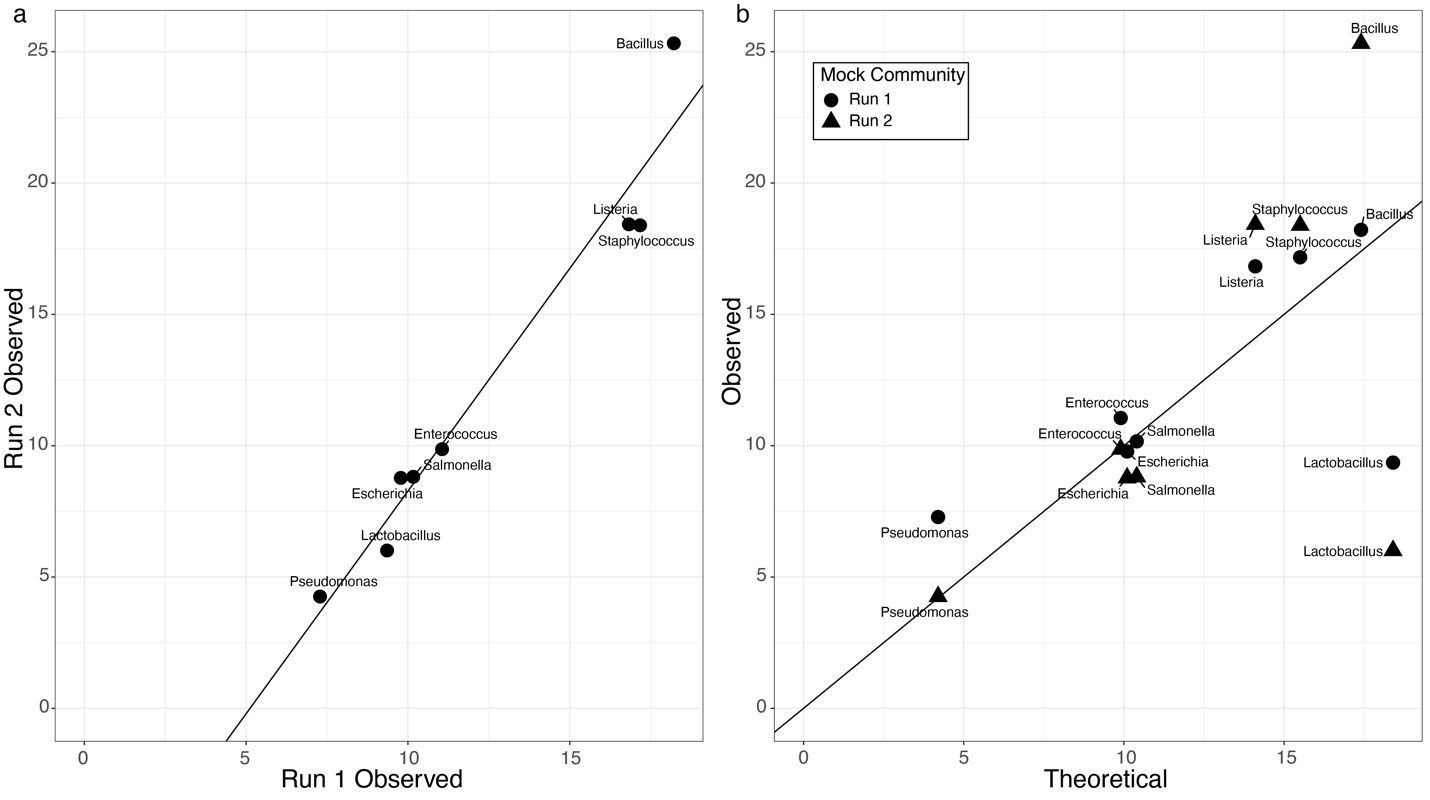
**Figure S2:** Results from a mock community that was co-analyzed with samples used in this study. a) A comparison of reproducibility between the observed relative abundance of taxa in the two sequencing runs that were used to generate data for this study, with the line representing a linear regression fit to the observed data (r^2^ = 0.95). b) A comparison of relative versus theoretical percent abundance in the library, with line representing a 1:1 ratio of observed to theoretical values. Not shown in b are the lines for the linear regression for run 1 and 2 (r^2^ = 0.40 and 0.35).


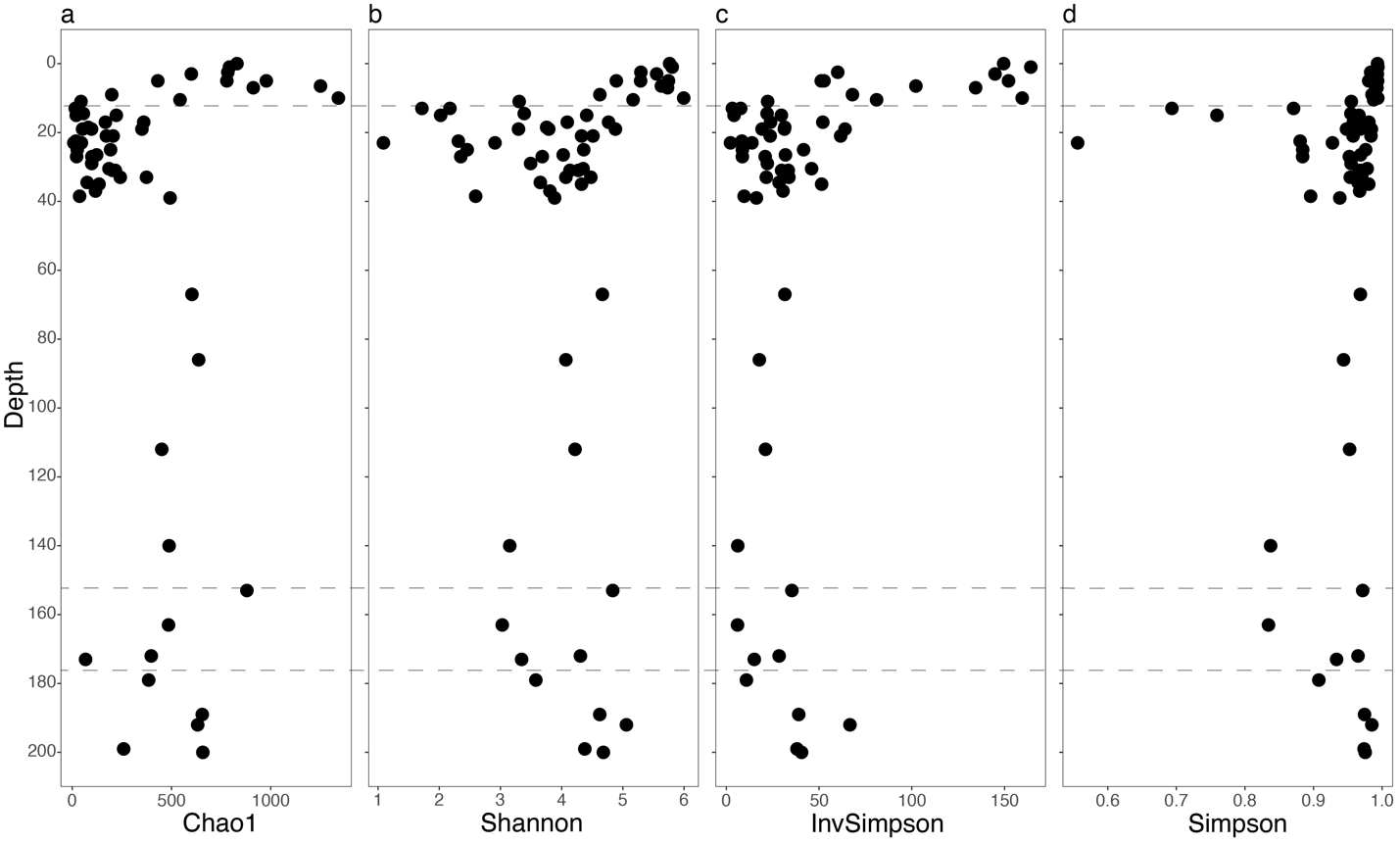
**Figure S3:** Downcore profiles of a) Chao1, b) Shannon Diversity, c) Inverse Simpson, and d) Simpson indices in the composite sediment profile. Dashed lines represent the boundaries between the stratigraphic units.


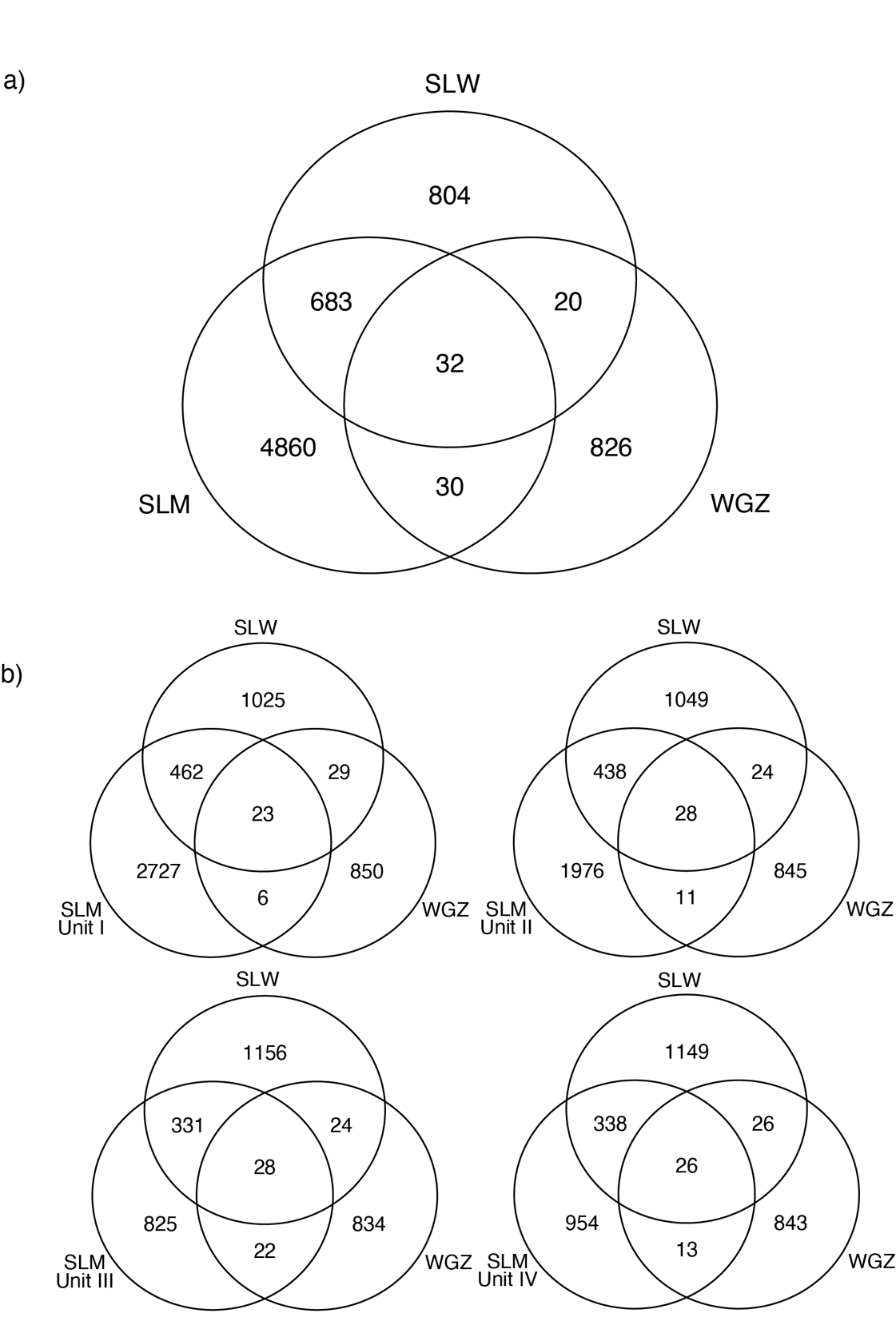
**Figure S4:** Venn diagrams showing distribution of ASVs in sediment samples from SLM, SLW, and the WGZ. Panel a is a bulk comparison, whereas panel b shows results for each unit in the SLM sediment profile.


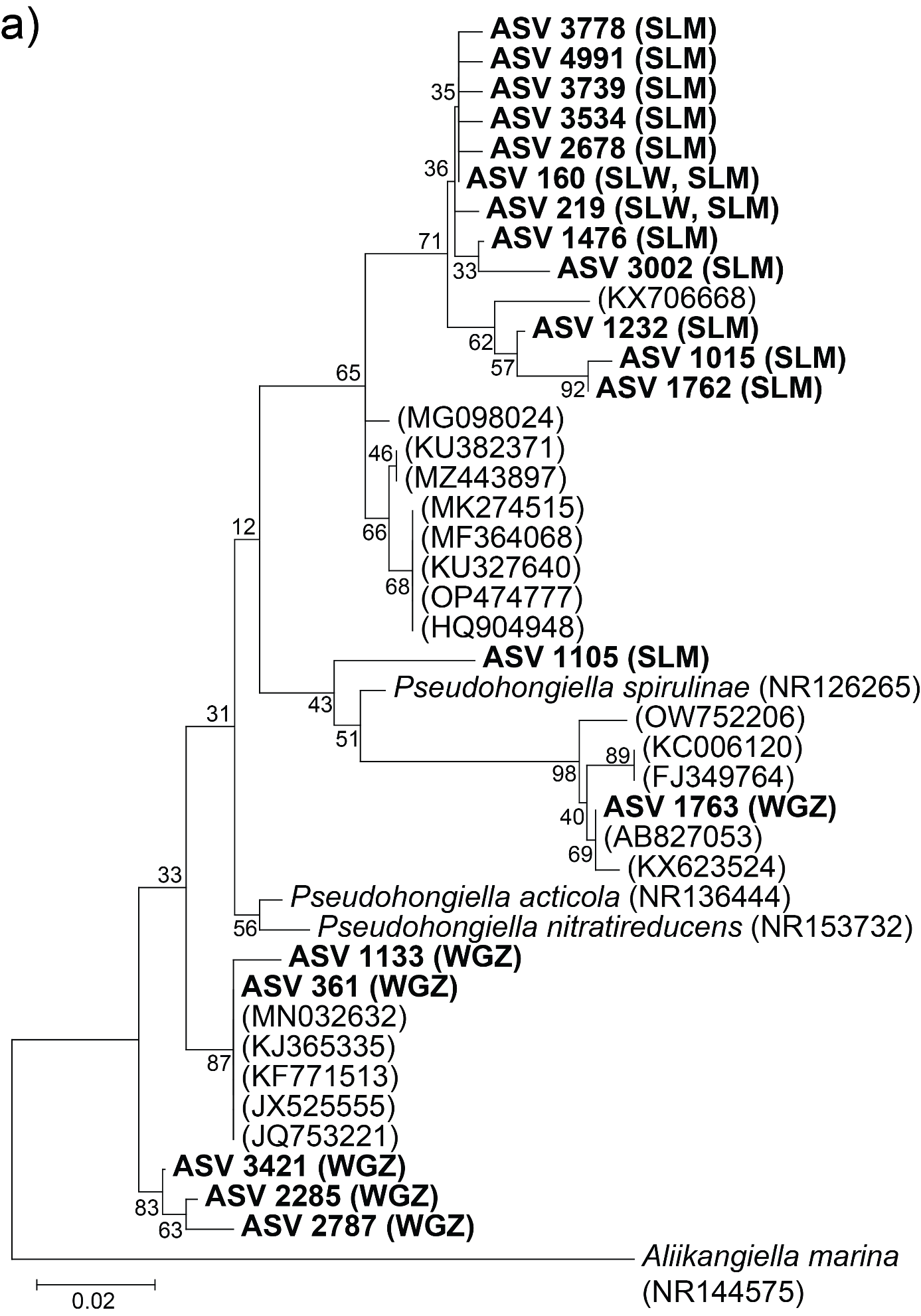


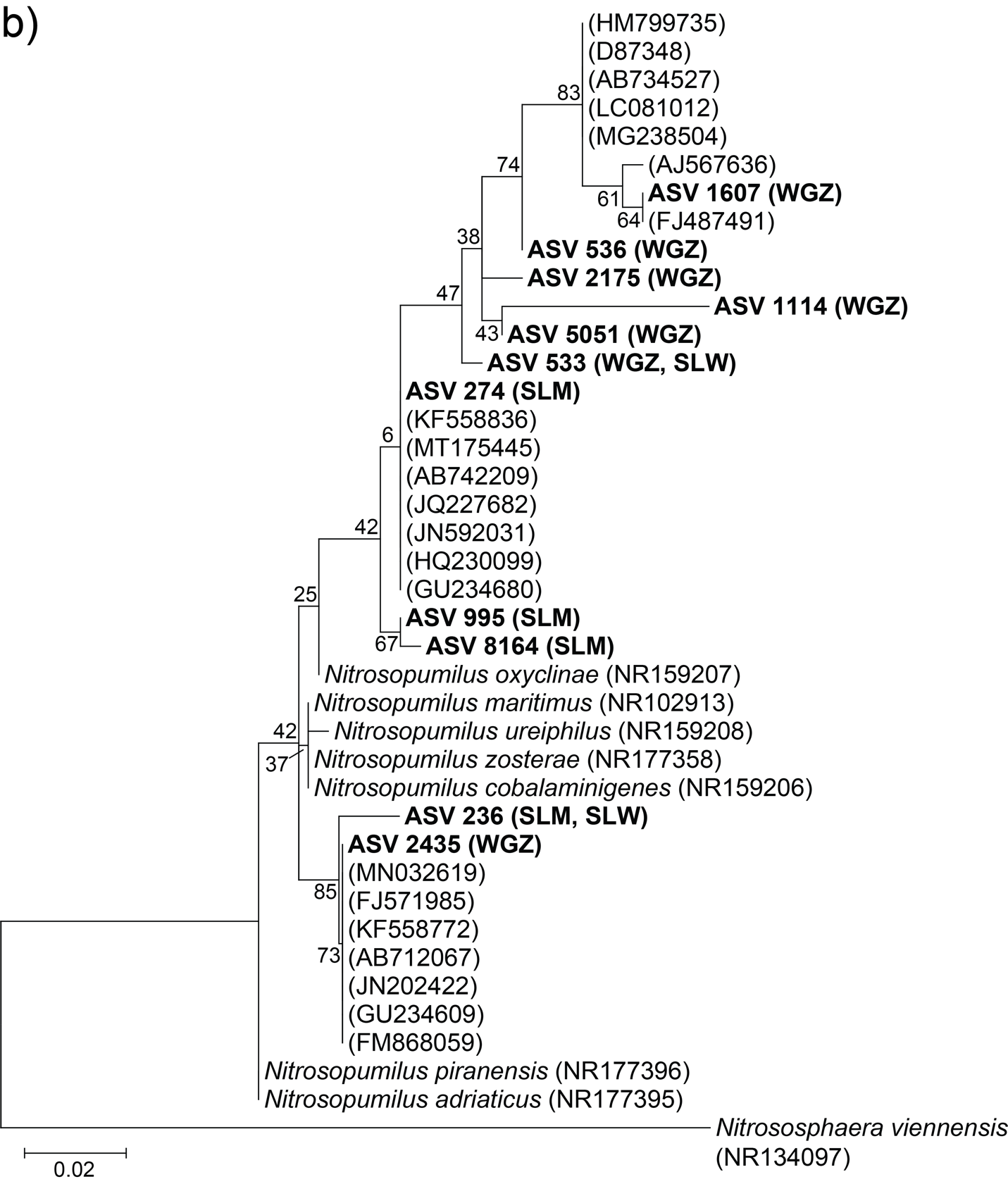


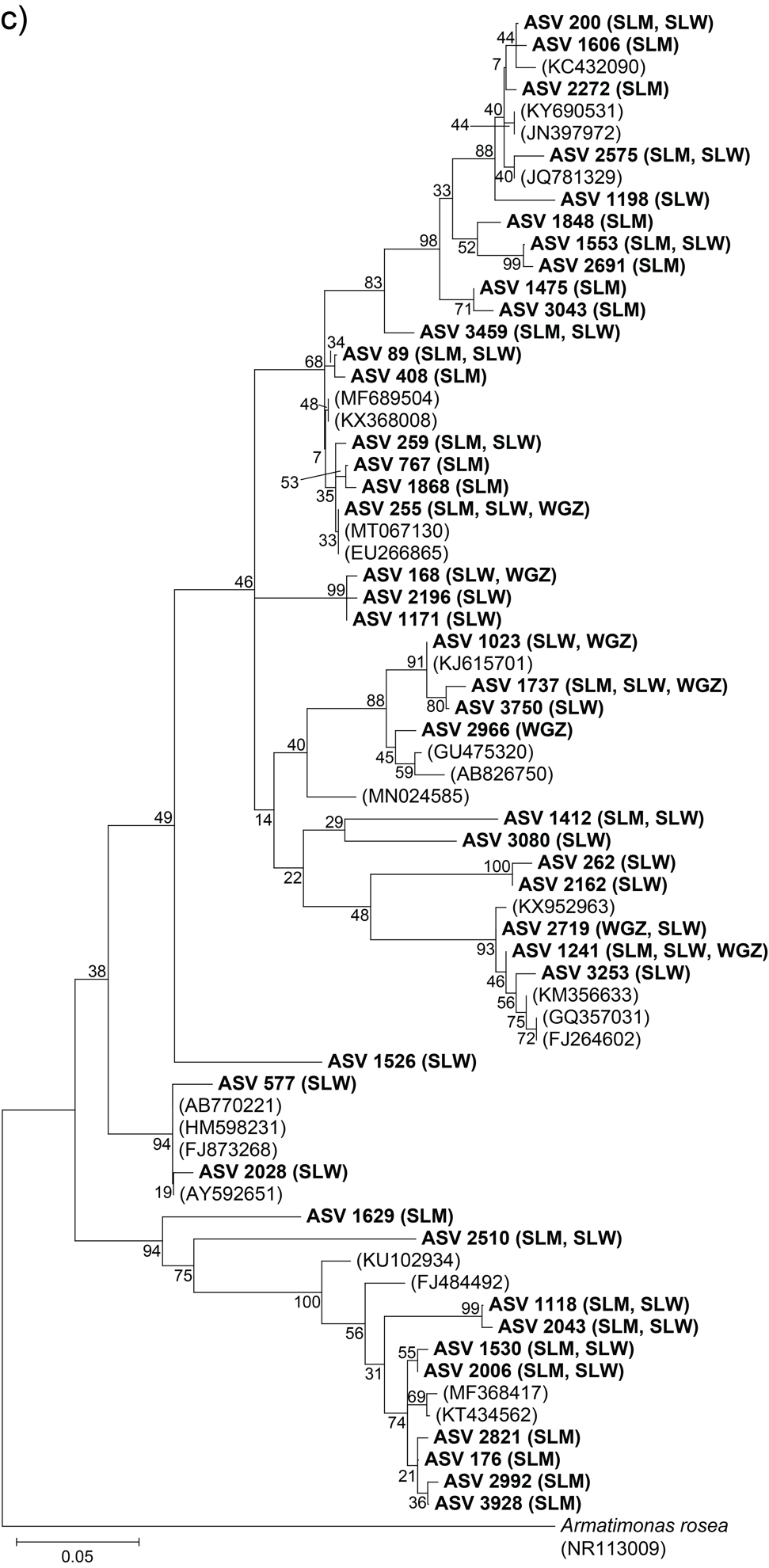


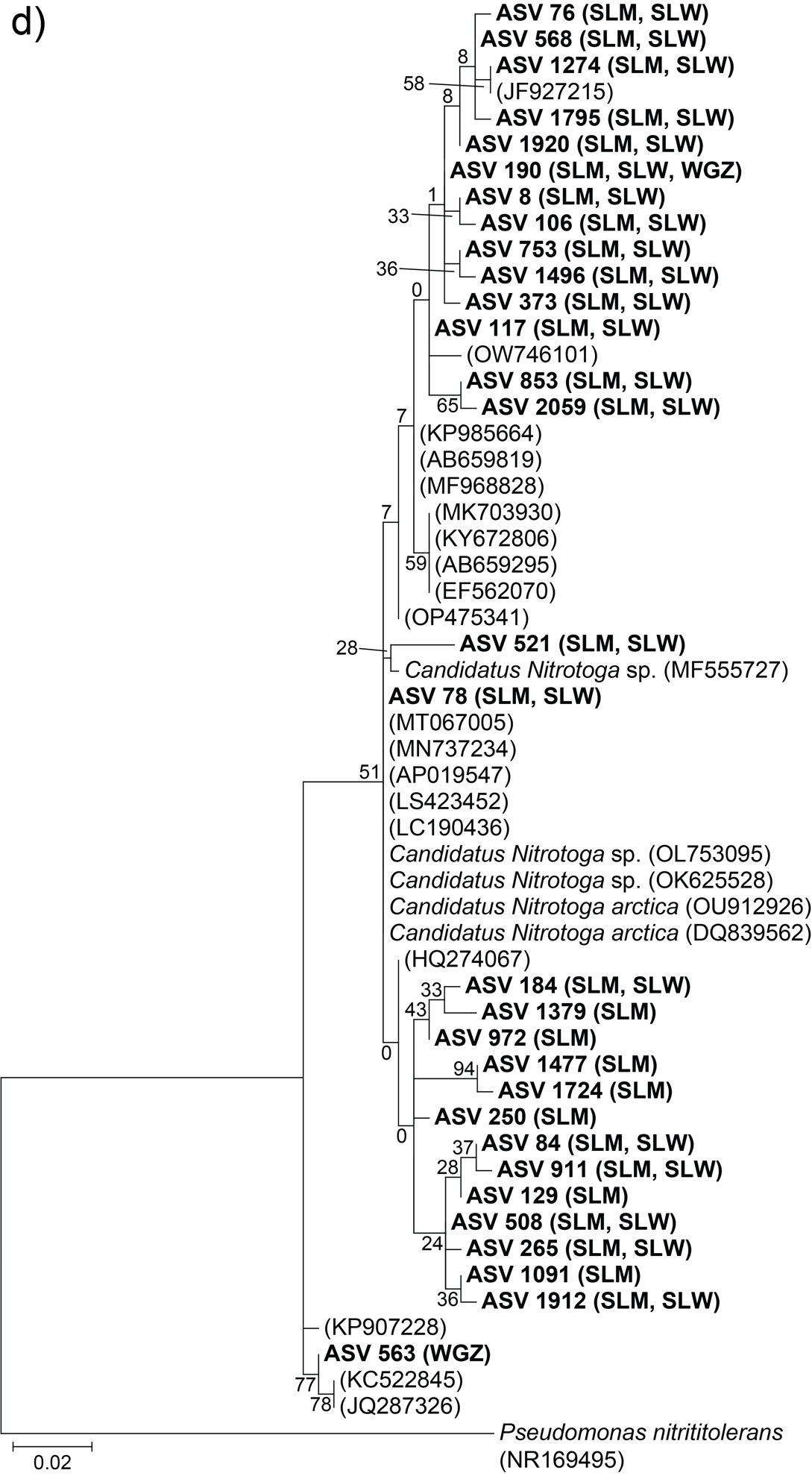


Figure S5: Maximum likelihood analysis based on alignment of partial 16S rRNA gene sequences from the ASVs shown in Figure 5b-e). The phylogenetic analysis was carried out with taxa affiliated with the class a) *Pseudohongiella* b) *Candidatus* Nitrosopumilus, c) Anaerolineaceae and genera, and d) *Candidatus* Nitrotoga. The scale bar represents the number fixed substitutions per nucleotide position. Bootstrap values are shown at the nodes and represent 100 replications.
